# Prospective validation of imaging and serum diagnostic biomarkers of steatohepatitis and fibrosis in MASLD: the LITMUS Imaging Study

**DOI:** 10.1038/s41591-026-04496-2

**Published:** 2026-07-24

**Authors:** Michael Pavlides, Yasaman Vali, Ferenc E. Mózes, Kristy Wonders, Salma Akhtar, Paul D. Hockings, Elizabeth Shumbayawonda, Kay Pepin, Isabel Fernandez-Lizaranzu, Diana Julie Leeming, Jeremy F. Cobbold, Michael E. D. Allison, Guruprasad P. Aithal, Manuel Romero-Gomez, Rocio Aller, Juan M. Pericàs, Jérôme Boursier, Salvatore Petta, Jörn M. Schattenberg, Mattias Ekstedt, Annalisa Berzigotti, Hannele Yki-Järvinen, Miljen Martic, Theresa Tuthill, Clifford A. Brass, Michael Kalutkiewicz, Rajarshi Banerjee, Richard L. Ehman, Moritz J. Schneider, Dina Tiniakos, Morten Karsdal, Jeremy Magnanensi, Céline Fournier-Poizat, Vlad Ratziu, Carla Yunis, Elisabetta Bugianesi, Stephen A. Harrison, LITMUS Consortium Investigators, Quentin M. Anstee, Ann Daly, Fiona Oakley, Simon J. Cockell, Dina Tiniakos, Pierre Bedossa, Heather J. Cordell, Christopher P. Day, Kristy Wonders, Olivier Govaere, Paolo Missier, Matthew McTeer, Luke Vale, Yemi Oliboyede, Matt Breckons, Patrick M. Bossuyt, Yasaman Vali, Jenny Lee, Max Nieuwdorp, Yair Acherman, Arnold van de Laar, Athanasios Angelakis, Adriaan G. Holleboom, Vlad Ratziu, Karine Clément, Rafael Patino-Navarrete, Raluca Pais, Valerie Paradis, Mathilde Wagner, Jörn M. Schattenberg, Maurice Michel, Detlef Schuppan, Sudha Myneni, Rambabu Surabattula, Beate K. Straub, Emrich Tilman, Toni Vidal-Puig, Sergio Rodrigues-Cuenca, Ioannis Kamzolas, Evangelia Petsalaki, Mark Campbell, Chris J. Lelliott, Michael E. D. Allison, Susan Davies, Edmund Godfrey, Michele Vacca, Matej Orešič, Aidan McGlinchey, Tuulia Hyötyläinen, Jose M. Mato, Óscar Millet, Annalisa Berzigotti, Jean-François Dufour, Naomi F. Lange, Adrian T. Huber, Michael Pavlides, Jeremy F. Cobbold, Stefan Neubauer, Ferenc E. Mozes, Salma Akhtar, Stephen A. Harrison, Seliat Olodo-Atitebi, Rajarshi Banerjee, Elizabeth Shumbayawonda, Andrea Dennis, Anneli Andersson, Manuel Romero-Gómez, Rocío Gallego-Durán, Rocío Montero-Vallejo, Isabel Fernandez-Lizarazu, Sheila Gato, Javier Castell, Morten Karsdal, Diana Julie Leeming, Kishwar Musa, Maria Manuela Tonini, Elisabetta Bugianesi, Chiara Rosso, Angelo Armandi, Ricardo Faletti, Fabio Marra, Amalia Gastaldelli, Fabrizia Carli, Gianluca Svegliati-Baroni, Jérôme Boursier, Marc de Saint Loup, Pierre Celea, Christophe Aube, Sven M. A. Francque, Wilhelmus J. Kwanten, Luisa Vonghia, An Verrijken, Eveline Dirinck, Ann Driessen, Mattias Ekstedt, Stergios Kechagias, Peter Lundberg, Hannele Yki-Järvinen, Kimmo Porthan, Johanna Arola, Saskia van Mil, George Papatheodoridis, Helena Cortez-Pinto, Mariana Verdelho Machado, Sofia Carvalhana, Cecilia M. P. Rodrigues, Luca V. C. Valenti, Serena Pelusi, Salvatore Petta, Grazia Penisi, Rosaria Maria Pipitone, Adele Tulone, Stefania Grimaudo, Michela Antonucci, Luca Miele, Antonio Liguori, Francesca D’Ambrosio, Andreas Geier, P. D. Monika Rau, Hans Benno Leicht, Florian Reiter, Guruprasad P. Aithal, Susan Francis, Naaventhan Palaniyappan, Christopher Bradley, Paul D. Hockings, Moritz J. Schneider, Philip N. Newsome, David Wenn, Jeremy Magnanensi, Aldo Trylesinski, Rebeca Mayo, Cristina Alonso, Kevin L. Duffin, James W. Perfield, Mark L. Hartman, Yu Chen, Carla Yunis, Theresa Tuthill, Trenton Ross, Barbara Bernardo, Magdalena Alicja Harrington, Euan McLeod, Judith Ertle, Ramy Younes, Harvey Coxson, Joseph Gogain, Hannah R. Biegel, Leigh Alexander, Rachel Ostroff, Mette Skalshøi Kjær, Lea Mørch Harder, Sanne S. Veidal, Naba Al-Sari, Daniel Guldager Kring Rasmussen, Maria-Magdalena Balp, Clifford A. Brass, Lori Jennings, Miljen Martic, Jurgen Loffler, Douglas Applegate, Richard Torstenson, Daniel Lindén, Céline Fournier-Poizat, Anne Llorca, Michael Kalutkiewicz, Richard L. Ehman, Kay Pepin, Gerald Horan, Gideon Ho, Dean Tai, Elaine Chng, Yayun Ren, Scott D. Patterson, Andrew N. Billin, Lynda C. Doward, James Twiss, Paresh Thakker, Zoltan Derdak, Henrik Landgren, Carolin Lackner, Annette S. H. Gouw, Prodromos Hytiroglou, Rocio Aller, Rebeca Sigüenza González, Juan M. Pericàs, Salvador Agustin, Jesús M. Rivera-Esteban, Sergio Muñoz-Martínez, Alba M. Jiménez-Masip, Laura Pagès, Diego Rojo, Patrick M. Bossuyt, Quentin M. Anstee

**Affiliations:** 1 Division of Cardiovascular Medicine, Radcliffe Department of Medicine, University of Oxford, Oxford, UK; 2 Translational Gastroenterology and Liver Unit, University of Oxford, Oxford, UK; 3 Oxford NIHR Biomedical Research Centre, Oxford University Hospitals NHS Foundation Trust and the University of Oxford, Oxford, UK; 4 Department of Epidemiology and Data Science, Amsterdam Public Health, Amsterdam UMC, University of Amsterdam, Amsterdam, The Netherlands; 5 Boehringer Ingelheim Pharma GmbH & Co. KG, Biberach, Germany; 6 Translational and Clinical Research Institute, Faculty of Medical Sciences, Newcastle University, Newcastle upon Tyne, UK; 7 Newcastle NIHR Biomedical Research Centre, Newcastle upon Tyne Hospitals NHS Foundation Trust, Newcastle upon Tyne, UK; 8 Antaros Medical AB, Mölndal, Sweden; 9 Perspectum Ltd., Oxford, UK; 10 Resoundant Inc., Rochester, MN USA; 11 Instituto de Biomedicina de Sevilla, IBiS/Hospital Universitario Virgen del Rocío/CSIC/Universidad de Sevilla, Sevilla, Spain; 12 Hepatic and Digestive Diseases Networking Biomedical Research Centre (CIBERehd), Grupo de Física Interdisciplinar, Universidad de Sevilla, Sevilla, Spain; 13 Nordic Bioscience, Biomarkers & Research, Herlev, Denmark; 14 Liver Unit, Department of Medicine, Cambridge NIHR Biomedical Research Centre, Cambridge University NHS Foundation Trust, Cambridge, UK; 15 NIHR Nottingham Biomedical Research Centre, Nottingham University Hospitals NHS Trust and the University of Nottingham, Nottingham, UK; 16 Nottingham Digestive Diseases Centre, Translational Medical Sciences, School of Medicine, University of Nottingham, Nottingham, UK; 17 Digestive Diseases Unit, Hospital Universitario Virgen del Rocío, Sevilla, Spain; 18 Hepatic and Digestive Diseases Networking Biomedical Research Centre (CIBERehd), Instituto de Biomedicina de Sevilla, Universidad de Sevilla, Sevilla, Spain; 19 Department of Gastroenterology, Clinic University Hospital, Medical School, University of Valladolid, CIBERINFEC, Valladolid, Spain; 20 Liver Unit, Vall d’Hebron Institut de Recerca, Vall d’Hebron Barcelona Hospital, Universitat Autònoma de Barcelona, Centros de Investigación Biomédica en Red Enfermedades Hepáticas y Digestivas (CIBERehd), Barcelona, Spain; 21 HIFIH Laboratory, SFR ICAT 4208, Angers University, Angers, France; 22 Hepato-Gastroenterology and Digestive Oncology Department, Angers University Hospital, Angers, France; 23 Section of Gastroenterology and Hepatology, PROMISE, Palermo, Italy; 24 Department of Internal Medicine II, Saarland University Medical Center, Homburg, Germany; 25 Department of Health, Medicine and Caring Sciences, Linköping University, Linköping, Sweden; 26 Department for Visceral Medicine and Surgery, Inselspital, Bern University Hospital, University of Bern, Bern, Switzerland; 27 Department of Biomedical Research, University of Bern, Bern, Switzerland; 28 Department of Medicine, University of Helsinki and Helsinki University Hospital, Helsinki, Finland; 29 Minerva Foundation Institute for Medical Research, Helsinki, Finland; 30 Novartis Institutes for BioMedical Research, Basel, Switzerland; 31 Digital Sciences and Translational Imaging, Pfizer Inc., Cambridge, MA USA; 32 Resolution Therapeutics, London, UK; 33 Department of Radiology, Mayo Clinic, Rochester, MN USA; 34 Department of Pathology, Aretaieion Hospital, Medical School, National and Kapodistrian University of Athens, Athens, Greece; 35 Genfit S.A., Loos, France; 36 Echosens, Paris, France; 37 ICAN Institute for Cardiometabolism and Nutrition, INSERM UMRS 1138 CRC, Sorbonne Université, Paris, France; 38 Assistance-Publique Hôpitaux de Paris, INSERM UMRS 1138 CRC, Paris, France; 39 Research and Development, Pfizer Inc., Lake Mary, FL USA; 40 Department of Medical Sciences, University of Turin, Torino, Italy; 41 Population Health Sciences Institute, Faculty of Medical Sciences, Newcastle University, International Centre for Life, Newcastle upon Tyne, UK; 42 Population Health Sciences Institute, Faculty of Medical Sciences, Newcastle University, Newcastle upon Tyne, UK; 43 Department of Surgery, Spaarne Hospital in Hoofddorp, Amsterdam, The Netherlands; 44 Department of Vascular Medicine, Amsterdam UMC, Amsterdam, The Netherlands; 45 INSERM UMRS 1269 Nutriomique, Sorbonne Université, Paris, France; 46 Department of Pathology, Beaujon hospital, APHP.Nord, Paris, France; 47 Radiology Department, AP-HP.6, GH Pitié Salpêtrière, Paris, France; 48 University Medical Center Mainz, Mainz, Germany; 49 Institute for Pathology, University Medicine Mainz, Johannes Gutenberg-University Mainz, Mainz, Germany; 50 Department of Diagnostic and Interventional Radiology, University Medical Center of Johannes Gutenberg-University, Mainz, Germany; 51 TVPLab, Metabolic Research Laboratories, Level 4, Institute of Metabolic Science, Addenbrooke’s Hospital, Cambridge, UK; 52 Department of Histopathology, Cambridge University Hospitals NHS Foundation Trust, Cambridge, UK; 53 Department of Radiology, Cambridge University NHS Foundation Trust, Cambridge, UK; 54 Interdisciplinary Department of Medicine, Università degli Studi di Bari “Aldo Moro”, Bari, Italy; 55 The Roger Williams Institute of Hepatology, Foundation for Liver Research, London, UK; 56 School of Medical Sciences, Faculty of Medicine and Health, Örebro University, Örebro, Sweden; 57 School of Science and Technology, Örebro University, Örebro, Sweden; 58 CIC bioGUNE, Derio, Spain; 59 Department of Diagnostic, Interventional and Pediatric Radiology, Inselspital, Bern University Hospital, University of Bern, Bern, Switzerland; 60 Translational Medicine Operations Hub, Luxembourg Institute of Health, Dudelange, Luxembourg; 61 Hepatology Laboratory, Department of Experimental and Clinical Medicine, Caregii Medical Clinic, Firenze, Italy; 62 Cardiometabolic Risk Unit, Institute of Clinical Physiology, CNR, Pisa, Italy; 63 Università Politecnica delle Marche, Responsabile Struttura Dipartimentale Danno Epatico e Trapianti, Ospedali Riuniti di Ancona, Ancona, Italy; 64 Department of Radiology, Centre Hospitalier Universitaire d’Angers, Angers, France; 65 Laboratoire HIFIH UPRES EA3859, Université d’Angers, Angers, France; 66 Department of Gastroenterology and Hepatology, Antwerp University Hospital, Edegem, Belgium; 67 Laboratory of Experimental Medicine and Paediatrics, InflaMed Centre of Excellence, Faculty of Medicine and Health Sciences, University of Antwerp, Wilrijk, Belgium; 68 Department of Pathology, Antwerp University Hospital, Edegem, Belgium; 69 Department of Radiation Physics, and Centre for Medical Image Science and Visualization (CMIV), Linköping University, Linköping, Sweden; 70 Faculty of Medicine, University of Helsinki, Helsinki, Finland; 71 Center for Molecular Medicine, UMC Utrecht, Utrecht, The Netherlands; 72 First Academic Department of Gastroenterology, Medical School of National and Kapodistrian University of Athens, General Hospital of Athens “Laiko”, Athens, Greece; 73 Clínica Universitária de Gastrenterologia, Faculdade de Medicina, Universidade de Lisboa, Lisbon, Portugal; 74 Research Institute for Medicines (iMed.ULIsboa), Faculty of Pharmacy, Universidade de Lisboa, Lisbon, Portugal; 75 Department of Pathophysiology and Transplantation, Università degli Studi di Milano, Milano, Italy; 76 Precision Medicine and Biological Resource Center, Fondazione IRCCS Ca’ Granda Ospedale Maggiore Policlinico Milano, Milano, Italy; 77 Section of Radiology—Di.Bi.Me.F., University of Palermo, Palermo, Italy; 78 Department of Translational Medicine and Surgery, Università Cattolica del Sacro Cuore and Fondazione Policlinico Gemelli IRCCS, Rome, Italy; 79 Division of Hepatology, Department of Medicine II, University Hospital Wuerzburg (UKW), Würzburg, Germany; 80 Sir Peter Mansfield Imaging Centre, Nottingham, UK; 81 Roger Williams Institute of Liver Studies, Faculty of Life Sciences and Medicine, King’s College London and King’s College Hospital, London, UK; 82 College of Medical and Dental Sciences, University of Birmingham, Birmingham, UK; 83 iXscient Ltd., London, UK; 84 Intercept Medical Head, Paris, France; 85 OWL Metabolomics, Derio, Spain; 86 Eli Lilly and Company, Indianapolis, IN USA; 87 Internal Medicine Research Unit, Pfizer Inc., Cambridge, MA USA; 88 Global Access & Value, Pfizer Inc., New York, NY USA; 89 Pfizer Ltd., Tadworth, UK; 90 TA CardioMetabolism Respiratory Medicine, Boehringer Ingelheim Pharma GmbH & Co. KG, Biberach, Germany; 91 Boehringer Ingelheim International GmbH, Ingelheim am Rhein, Germany; 92 SomaLogic, Inc., Boulder, CO USA; 93 Clinical Development, Novo Nordisk A/S, Søborg, Denmark; 94 Global Drug Discovery, Novo Nordisk, Maaloev, Denmark; 95 Computational Biomarker Discover Department, DSI, Måløv, Denmark; 96 Novo Nordisk, Søborg, Denmark; 97 Novartis Pharma AG, Basel, Switzerland; 98 Regulatory Affairs, Cardiovascular, Renal and Metabolism (CVRM), BioPharmaceuticals R&D, AstraZeneca, Gothenburg, Sweden; 99 Bristol-Myers Squibb, Bothell, FL USA; 100 Histoindex, Singapore, Singapore; 101 Gilead Sciences, Foster City, CA USA; 102 European Head, Patient-Centered Outcomes Assessment, RTI Health Solutions, Manchester, UK; 103 Gastrointestinal and Inflammation Therapeutic Area Unit Takeda Development Centers of America, Cambridge, MA USA; 104 AbbVie Inc., North Chicago, IL USA; 105 Institute of Pathology, Medical University of Graz, Graz, Austria; 106 Department of Pathology and Medical Biology, University Medical Center Groningen, Groningen, The Netherlands; 107 Department of Pathology, Aristotle University School of Medicine, Thessaloniki, Greece; 108 Department of Radiology, Clinic University Hospital, Medical School, University of Valladolid, Valladolid, Spain

**Keywords:** Diagnostic markers, Non-alcoholic steatohepatitis

## Abstract

There is a need for robust evaluations of noninvasive biomarkers for metabolic dysfunction-associated steatotic liver disease. This prospective multicenter study assessed the diagnostic accuracy of imaging (including liver stiffness measurement (LSM) using magnetic resonance elastography (MRE) and FibroScan (vibration-controlled transient elastography (VCTE)), serum biomarkers (including NIS2+) and composite scores (including Agile 3+ and Agile 4) for centrally read steatohepatitis and fibrosis. For cirrhosis, several biomarkers exceeded the minimum acceptable performance criterion (MAC), including MRE (area under the receiver operating curve (AUC) 0.91; *P* < 0.01), VCTE-LSM (AUC 0.87; *P* < 0.01), Agile 3+ (AUC 0.89; *P* < 0.01) and Agile 4 (AUC 0.88; *P* < 0.01). Among 357 participants, fibrosis stages F0–F4 were present in 12%, 16%, 25%, 32% and 15%, respectively. NIS2+ had the highest diagnostic accuracy among serum biomarkers for metabolic dysfunction-associated steatohepatitis (MASH; AUC 0.83) and at-risk MASH (MASH with at least stage 2 fibrosis; AUC 0.82), although neither significantly exceeded the MAC (*P* = 0.15 and *P* = 0.19). Among imaging biomarkers, MRE showed the highest performance for MASH (AUC 0.71) and at-risk MASH (AUC 0.75), but these remained below the MAC. For fibrosis staging, performance was stronger: MRE met the MAC for advanced fibrosis (AUC 0.91; *P* < 0.01), as did Agile 3+ (AUC 0.84; *P* = 0.03). For cirrhosis, several biomarkers exceeded the MAC, including MRE (AUC 0.91; *P* < 0.01), VCTE-LSM (AUC 0.87; *P* < 0.01), Agile 3+ (AUC 0.89; *P* < 0.01) and Agile 4 (AUC 0.88; *P* < 0.01). Serum biomarkers tended to outperform imaging for identifying at-risk MASH, whereas elastography and composite scores showed excellent accuracy for staging advanced fibrosis and cirrhosis.

## Abstract (web summary)

In the LITMUS study, serum biomarkers outperformed imaging for identifying patients at risk of MASH, whereas elastography was superior for staging advanced fibrosis and cirrhosis.

## Main

Metabolic dysfunction-associated steatotic liver disease (MASLD), formerly known as nonalcoholic fatty liver disease, is the most common cause of chronic liver disease worldwide^1,2^. MASLD affects 25%–30% of people in the general population, and in those with obesity and type 2 diabetes mellitus (T2DM), the prevalence of MASLD can be up to 70% (ref. ^2^). MASLD includes a wide range of pathological conditions ranging from the accumulation of fat only (isolated steatosis, MASL) to the accumulation of fat with inflammation and liver cell damage (hepatocyte ballooning), collectively termed metabolic dysfunction-associated steatohepatitis (MASH) with increasing stages of fibrosis up to cirrhosis (F0–4)^3^. Progressive disease, with greater fibrosis or cirrhosis, is associated with increasing risk of adverse clinical outcomes, including hepatic decompensation and development of hepatocellular carcinoma^4–6^.

The first pharmacotherapy for pre-cirrhotic MASH with significant or advanced fibrosis (F2–3) recently received US Food and Drug Administration (FDA) approval, and numerous other agents are being evaluated in clinical trials^7,8^. Beyond the well-documented challenges of using histology for patient selection and evaluation of treatment response in clinical trials^9–11^, the advent of licensed drug therapies emphasizes the need for scalable diagnostics that can safely and accurately guide patient selection for treatment in routine care^12^. Liver biopsy is resource intensive. It necessitates procedural expertise, facilities for patient monitoring post-biopsy, laboratory facilities for biopsy sample processing and expert histopathologists to read the biopsy results^13^. It is also an invasive procedure that carries the risk of procedure-related complications, ranging from common and mild (for example, pain following 30%–50% of procedures)^14^ to uncommon but potentially life threatening (serious hemorrhage occurs in 0.6% of procedures; estimated procedure-related mortality of 0.1% (ref. ^13^)). For these reasons, many patients are unwilling to undergo the procedure. There is an expectation that noninvasive tests will be used in routine care to select individuals for treatment and to assess whether treatment is effective and should be continued. The need for noninvasive biomarkers for MASH and fibrosis is, therefore, well established^12^.

A wide range of biomarkers based on serum or imaging tests, including elastography or combinations of the two, have been described. Vibration-controlled transient elastography (VCTE) is one of the most extensively studied, while magnetic resonance elastography (MRE) and other quantitative magnetic resonance (MR) techniques that do not require additional hardware have also been proposed. While a number of studies have evaluated individual biomarkers or a limited number of biomarkers in small cohorts, evaluation and validation of multiple imaging and blood-based biomarkers in a large, prospective multicenter study is lacking. Our study specifically addresses this important evidence gap: presenting prospectively acquired, robust and impartial data defining the diagnostic performance of multiple imaging and circulating biomarkers—encompassing tests that are well established and also cutting-edge technologies that are less widely available. The main aim of the Liver Investigation: Testing Marker Utility for Steatohepatitis (LITMUS) Imaging Study was to robustly assess and independently evaluate the performance of MR, ultrasound-based imaging and elastography biomarkers for the accurate grading and staging of MASLD. A secondary aim was to compare the performance of imaging biomarkers with that of serum biomarkers. This study was specifically designed to assess the diagnostic accuracy of noninvasive biomarkers in people with MASLD, who have already been referred to secondary or tertiary care, and the results should not be extrapolated to primary care population screening, in which disease prevalence will be lower.

## Results

### Characteristics of the LITMUS Imaging Study analysis set

**Extended Data Fig. 1:**
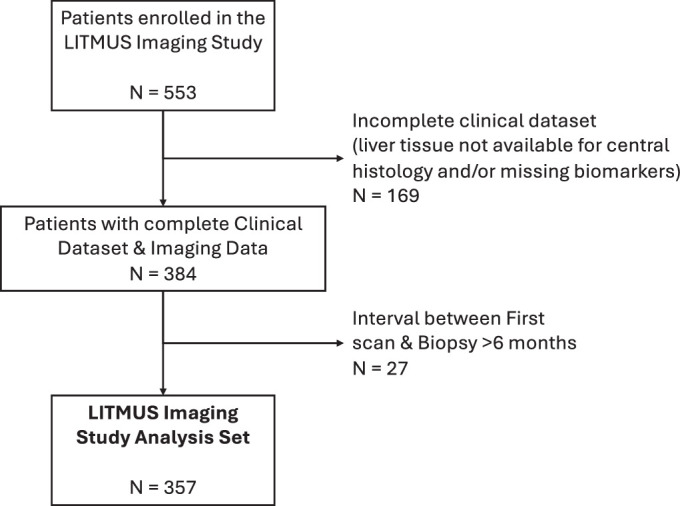
Study Consort Diagram. 553 patients took part in the LITMUS Imaging Study and had at least one imaging assessment. Histology was centrally read and serum biomarker results from the LITMUS central lab were both available for 384 of the participants in the LITMUS Imaging Study. Imaging and biopsy were not performed within 6 months of each other in 27 cases and these were excluded from analysis, leaving 357 cases as the study analysis set.

Within the LITMUS study cohort, 553 participants were enrolled in the LITMUS Imaging Study. Among these participants, 357 had both central biopsy reading and imaging data available with an interval of less than 6 months between their first scan and biopsy (Extended Data Fig. 1). Their characteristics were consistent with the spectrum of MASLD encountered in secondary and tertiary care settings: mean age, 55.4 years; male sex, 196 (55%); with white ethnicity, 335 (94%); mean body mass index (BMI), 33.8 kg m^−2^; with type 2 diabetes, 177 (50%); and with hypertension, 200 (56%) (Table 1 and Supplementary Table 1, showing the countries of recruitment).

**Table 1:** LITMUS Imaging Study analysis—participant characteristics

	Total (*n* = 357)	At-risk MASH (*n* = 164)	MASH (*n* = 182)	Advanced fibrosis (*n* = 166)	Cirrhosis (*n* = 51)
Age (years)	55.4 (13.2)	57.7 (12.2)	57.1 (12.3)	60.3 (10.5)	61.40 (7.4)
Sex (male; *n* (%))	196 (54.9)	87 (53.1)	96 (52.8)	91 (54.8)	29 (56.9)
Ethnicity (white; *n* (%))	335 (93.8)	156 (95.1)	170 (93.4)	155 (93.4)	46 (90.2)
BMI (mean (s.d.))	33.84 (5.6)	34.30 (5.5)	34.32 (5.5)	33.89 (5.32	33.92 (4.9)
Type 2 diabetes (*n* (%))	177 (49.6)	99 (60.3)	107 (58.8)	115 (69.3)	38 (74.5)
Hypertension (*n* (%))	200 (56.0)	99 (60.4)	108 (59.3)	111 (66.9)	32 (62.7)
ALT (U l^−1^; mean (s.d.))	57.2 (45.3)	65.3 (42.7)	66.8 (46.2)	53.5 (36.9)	53.5 (37.9)
AST (U l^−1^; mean (s.d.))	45.1 (27.6)	54.0 (30.3)	54.2 (30.6)	48.3 (28.0)	51.4 (29.6)
GGT (U l^−1^; mean (s.d.))	96.6 (133.4)	103.7 (101.5)	101.0 (98.5)	113.7 (143.6)	146.2 (133.4)
Albumin (g dl^−1^; mean (s.d.))	4.3 (0.4)	4.3 (0.4)	4.3 (0.4)	4.2 (0.5)	4.2 (0.6)
Glucose (mmol l^−1^; mean (s.d.))	6.8 (2.9)	7.7 (3.45)	7.6 (3.43)	7.7 (3.41)	8.2 (4.08)
Total cholesterol (mmol l^−1^; mean (s.d.))	4.7 (1.23)	4.8 (1.20)	4.7 (1.20)	4.5 (1.19)	4.9 (1.18)
Triglyceride (mmol l^−1^; mean (s.d.))	2.0 (1.15)	2.1 (1.17)	2.1 (1.14)	2.1 (1.30)	2.0 (1.28)
Platelet (10^9^ l^−1^; mean (s.d.))	224.1 (64.9)	221.1 (65.6)	221.8 (63.5)	208.2 (65.13)	182.6 (66.0)
Histology					
Steatosis (%)					
0	36 (10.1%)	–	–	9 (5.4%)	5 (9.8%)
1	114 (31.9%)	27 (16.5%)	28 (15.4%)	54 (32.5%)	19 (37.3%)
2	116 (32.5%)	71 (43.3%)	80 (44.0%)	62 (37.3%)	19 (37.3%)
3	91 (25.5%)	66 (40.2%)	74 (40.7%)	41 (24.7%)	8 (15.7%)
Inflammation (%)					
0	46 (12.9%)	–	–	4 (2.4%)	1 (2.0%)
1	236 (66.1%)	106 (64.6%)	123 (67.6%)	112 (67.5%)	41 (80.4%)
2	67 (18.8%)	50 (30.5%)	51 (28.0%)	43 (25.9%)	8 (15.7%)
3	8 (2.2%)	8 (4.9%)	8 (4.4%)	7 (4.2%)	1 (2.0%)
Ballooning (%)					
0	140 (39.2%)	–	–	27 (16.3%)	8 (15.7%)
1	124 (34.7%)	77 (47.0%)	89 (48.9%)	67 (40.4%)	20 (39.2%)
2	93 (26.1%)	87 (53.0%)	93 (51.1%)	72 (43.4%)	23 (45.1%)
Fibrosis stage (%)					
0	43 (12.0%)	–	0 (0%)	–	–
1	58 (16.2%)	–	18 (9.9%)	–	–
2	90 (25.2%)	47 (28.7%)	47 (25.8%)	–	–
3	115 (32.2%)	82 (50.0%)	82 (45.1%)	115 (69.3%)	–
4	51 (14.3%)	35 (21.3%)	35 (19.2%)	51 (30.7%)	51 (100%)
MAS (%)					
0	23 (6.4%)	–	–	2 (1.2%)	1 (2.0%)
1	27 (7.6%)	–	–	5 (3.0%)	2 (3.9%)
2	36 (10.1%)	–	–	9 (5.4%)	5 (9.8%)
3	69 (19.3%)	–	–	29 (17.5%)	7 (13.7%)
4	70 (19.6%)	48 (29.3%)	53 (29.1%)	39 (23.5%)	14 (27.5%)
5	73 (20.4%)	58 (35.4%)	70 (38.5%)	40 (24.1%)	14 (27.5%)
6	43 (12.0%)	42 (25.6%)	43 (23.6%)	31 (18.7%)	8 (15.7%)
7	14 (3.9%)	14 (8.5%)	14 (7.7%)	9 (5.4%)	0 (0%)
8	2 (0.6%)	2 (1.2%)	2 (1.1%)	2 (1.2%)	0 (0%)
MASH	182 (52.0%)	164 (100%)	182 (100%)	117 (70.5%)	35 (68.6%)

Continuous variables are shown as mean (s.d.); categorical variables are shown as number (%).

ALT, alanine aminotransferase; AST, aspartate aminotransferase; GGT, gamma glutamyl transferase.

Even though a maximum 6-month interval was allowable between liver biopsy and biomarker evaluation, the majority of participants had assessments within a shorter interval (82% and 75% of participants had imaging and blood-based biomarker acquisition, respectively, within 12 weeks of liver biopsy). The mean interval between biopsy and blood sampling and imaging assessment was 3.6 weeks (s.d. 4.8) and 7.0 weeks (s.d. 5.1), respectively. MASH was detected in 182 (51%) participants, with all fibrosis stages represented: F0: 43 (12%); F1: 58 (16%); F2: 90 (25%); F3: 115 (32%); and F4: 51 (15%). At-risk MASH (MASH with at least stage 2 fibrosis) was detected in 164 (46%) participants.

### Diagnostic performance for detecting MASH and at-risk MASH

**Extended Data Fig. 2:**
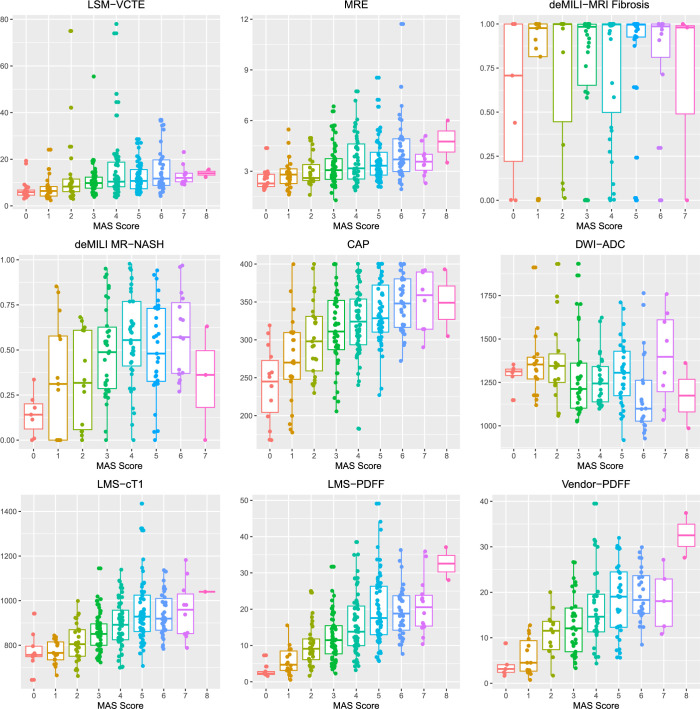
Distribution of imaging marker results by MAS score (0-8). Boxplots represent the distribution of individual imaging biomarker values stratified by MAS score (0–8). Boxes show the interquartile range with medians marked by horizontal lines; whiskers extend to 1.5× the IQR. Dots indicate observation from individual participants. Sample sizes vary across biomarkers: MRE (n = 275), LMS-cT1 (n = 265), LMS-PDFF (n = 304), Vendor-PDFF (n = 163), deMILI Fibrosis-MRI (n = 158), deMILI NASH-MRI (n = 158), DWI-ADC (n = 160), VCTE-LSM (n = 298), CAP (n = 267).

**Extended Data Fig. 3:**
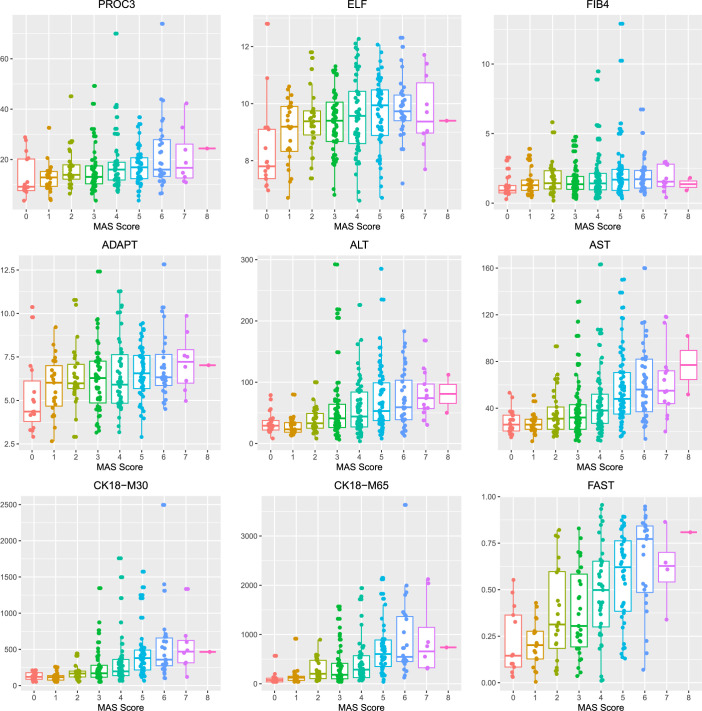
Distribution of serum marker results by MAS score (0-8). Boxplots represent the distribution of individual biomarker values stratified by MAS score (0–8). Boxes show the interquartile range with medians marked by horizontal lines; whiskers extend to 1.5× the IQR. Dots indicate observation from individual participants. Sample sizes vary across biomarkers: PRO-C3 (n = 250), ALT (n = 335), AST (n = 335), CK18-M30 (n = 239), CK18-M65 (n = 236), FIB-4 (n = 331), ADAPT (n = 246), ELF (n = 276), NIS2+ (n = 221).

**Extended Data Fig. 4:**
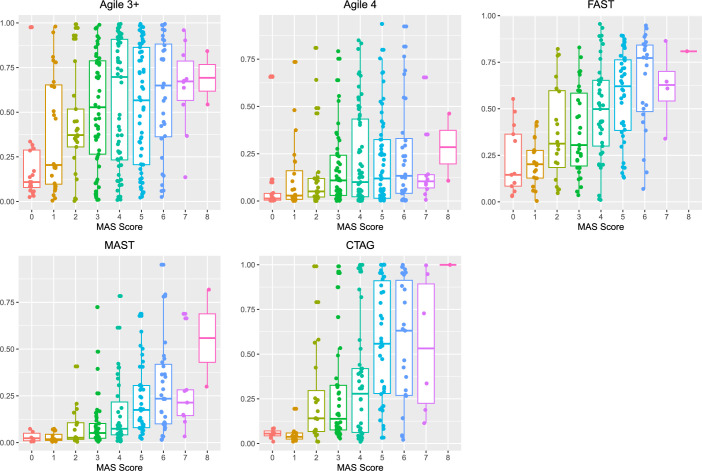
Distribution of combination marker results by MAS score (0-8). Boxplots represent the distribution of individual biomarker values stratified by MAS score (0–8). Boxes show the interquartile range with medians marked by horizontal lines; whiskers extend to 1.5× the IQR. Dots indicate observation from individual participants. Sample sizes vary across biomarkers: FAST (n = 190), Agile 3+ (n = 288), Agile 4 (n = 288), CTAG (n = 187), and MAST (n = 203).

**Extended Data Fig. 5:**
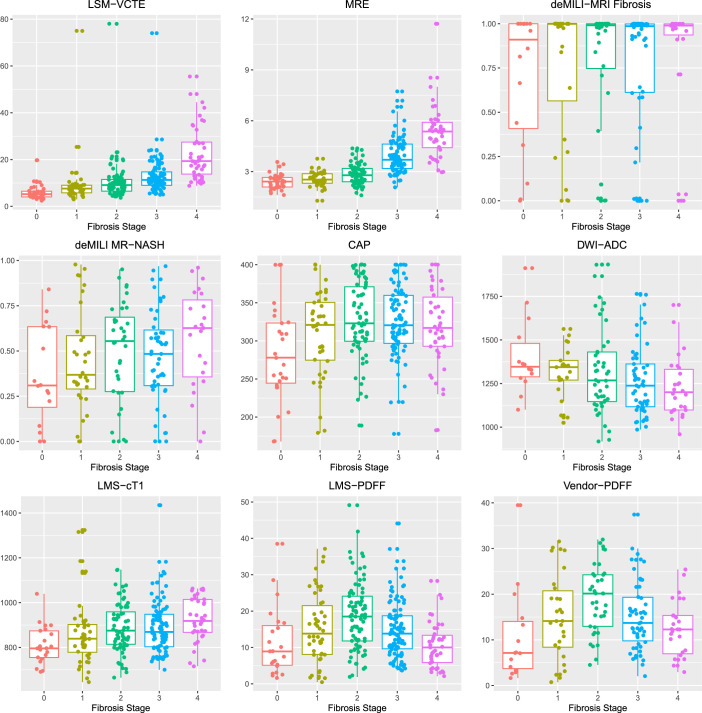
Distribution of imaging marker results by fibrosis stage (0-4). Boxplots represent the distribution of individual biomarker values stratified by Fibrosis stage (F0–4). Boxes show the interquartile range with medians marked by horizontal lines; whiskers extend to 1.5× the IQR. Dots indicate observation from individual participants. Sample sizes vary across biomarkers: MRE (n = 275), LMS-cT1 (n = 265), LMS-PDFF (n = 304), Vendor-PDFF (n = 163), deMILI Fibrosis-MRI (n = 158), deMILI NASH-MRI (n = 158), DWI-ADC (n = 160), VCTE-LSM (n = 298), CAP (n = 267).

**Extended Data Fig. 6:**
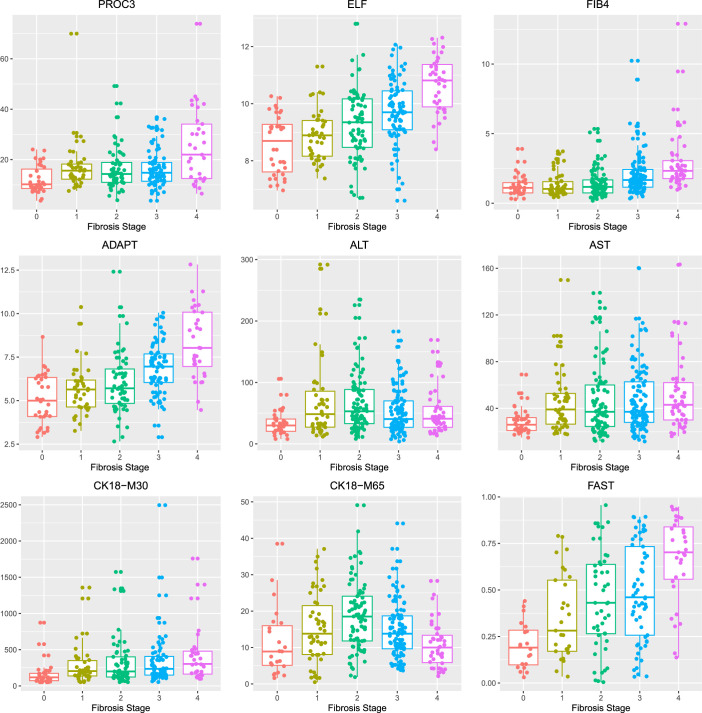
Distribution of serum marker results by fibrosis stage (0-4). Boxplots represent the distribution of individual biomarker values stratified by Fibrosis stage (F0–4). Boxes show the interquartile range with medians marked by horizontal lines; whiskers extend to 1.5× the IQR. Dots indicate observation from individual participants. Sample sizes vary across biomarkers: PRO-C3 (n = 250), ALT (n = 335), AST (n = 335), CK18-M30 (n = 239), CK18-M65 (n = 236), FIB-4 (n = 331), ADAPT (n = 246), ELF (n = 276), NIS2+ (n = 221).

**Extended Data Fig. 7:**
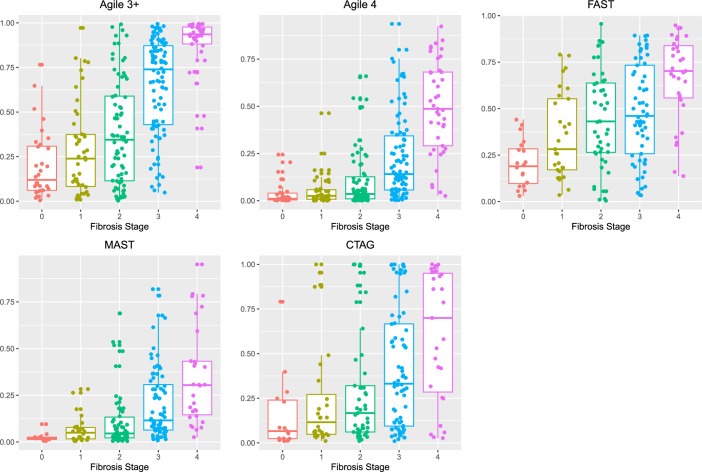
Distribution of combination marker results by fibrosis stage (0-4). Boxplots represent the distribution of individual biomarker values stratified by Fibrosis stage (F0–4). Boxes show the interquartile range with medians marked by horizontal lines; whiskers extend to 1.5× the IQR. Dots indicate observation from individual participants. Sample sizes vary across biomarkers: FAST (n = 190), Agile 3+ (n = 288), Agile 4 (n = 288), CTAG (n = 187), and MAST (n = 203).

Box plots of biomarker results plotted against the histological MASLD activity score (MAS) and fibrosis stage are shown in Extended Data Figs. 2–7.

**Fig. 1:**
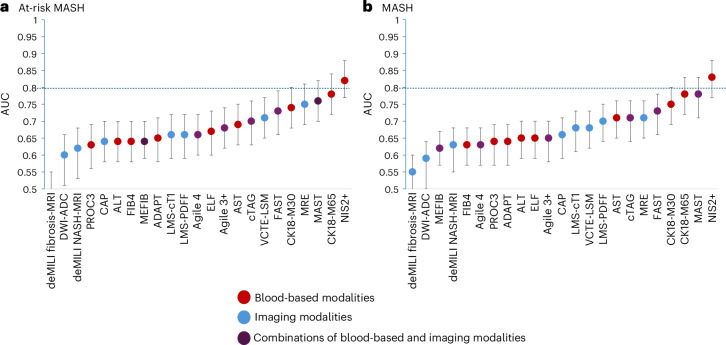
Diagnostic performance of biomarkers for MASH and at-risk MASH. Each point in this figure represents the AUC estimate for a marker, and error bars indicate 95% CIs for the AUC. Biomarkers are grouped by modality (blood-based, imaging and combination markers). The unit of analysis was the individual participant. AUC estimates and corresponding 95% CIs were derived from patient-level data. A dashed line to indicate the a priori-defined MAC of the AUC of 0.80 is included in each graph. Sample sizes varied across biomarkers as follows: MRE (*n* = 275), LMS-cT1 (*n* = 265), LMS-PDFF (*n* = 304), vendor-PDFF (*n* = 163), deMILI fibrosis-MRI (*n* = 158), deMILI NASH-MRI (*n* = 158), DWI-ADC (*n* = 160), VCTE-liver stiffness measurement by vibration controlled transient elastography (LSM) (*n* = 298), CAP (*n* = 267), N-terminal pro-peptide of type III collagen (PRO-C3) (*n* = 250), ALT (*n* = 335), AST (*n* = 335), CK18-M30 (*n* = 239), cytokeratin 18 measured using the M65 assay (CK18-M65) (*n* = 236), FIB-4 (*n* = 331), ADAPT (*n* = 246), ELF (*n* = 276), NIS2+ (*n* = 221), FAST (*n* = 190), Agile 3+ (*n* = 288), Agile 4 (*n* = 288), cTAG (*n* = 187) and MAST (*n* = 203). **a**, At-risk MASH: the highest AUCs for blood-based, imaging and combination markers were, respectively, 0.82 (0.77–0.88) for NIS2+, 0.75 (0.69–0.81) for MRE and 0.78 (0.71–0.84) for MAST. No marker exceeded the MAC. **b**, MASH: the highest AUCs for blood-based, imaging and combination markers were, respectively, 0.83 (0.77–0.88) for NIS2+, 0.71 (0.65–0.77) for MRE and 0.78 (0.71–0.84) for MAST. No marker exceeded the MAC.

Table 2 includes data for the performance of all the biomarkers for all four target conditions. The area under the receiver operating curve (AUC) for MASH or at-risk MASH ranged from 0.59 to 0.83. No biomarker significantly exceeded the minimum acceptable performance criterion (MAC) of an AUC of ≥0.80 for these two target conditions (Fig. 1).

**Table 2:** Diagnostic performance of all markers in detecting at-risk MASH, MASH, advanced fibrosis and cirrhosis

Biomarker	*n*	Target condition											
		At-risk MASH			MASH			Advanced fibrosis			Cirrhosis		
		%	AUC (95% CI)	*P* value	%	AUC (95% CI)	*P* value	%	AUC (95% CI)	*P* value	%	AUC (95% CI)	*P* value
**MRE**	275	47	0.75 (0.69–0.81)	0.96	51	0.71 (0.65–0.77)	1.00	46	0.91 (0.88–0.95)	0.00	15	0.91 (0.86–0.95)	0.00
**LMS-cT1**	265	46	0.66 (0.59–0.72)	1.00	52	0.68 (0.61–0.74)	1.00	50	0.59 (0.52–0.66)	1.00	14	0.64 (0.55–0.73)	1.00
**LMS-PDFF**	304	48	0.66 (0.59–0.72)	1.00	54	0.70 (0.64–0.76)	1.00	49	0.58 (0.52–0.65)	1.00	14	0.67 (0.59–0.75)	1.00
**Vendor-PDFF**	163	44	0.59 (0.51–0.68)	1.00	52	0.67 (0.58–0.75)	1.00	50	0.56 (0.47–0.65)	1.00	17	0.62 (0.51-0.72)	1.00
**deMILI Fibrosis-MRI**	158	41	0.49 (0.40–0.58)	1.00	50	0.55 (0.46–0.64)	1.00	47	0.55 (0.46–0.64)	1.00	15	0.49 (0.37–0.61)	1.00
**deMILI NASH-MRI**	158	41	0.62 (0.53–0.71)	1.00	50	0.63 (0.55–0.72)	1.00	47	0.55 (0.46–0.64)	1.00	15	0.62 (0.49–0.74)	1.00
**DWI-ADC**	160	47	0.60 (0.51–0.69)	1.00	49	0.59 (0.50–0.68)	1.00	49	0.62 (0.53–0.71)	1.00	16	0.62 (0.50–0.73)	1.00
**VCTE-LSM**	298	48	0.71 (0.65–0.77)	1.00	52	0.68 (0.62–0.75)	1.00	47	0.81 (0.76–0.86)	0.38	14	0.87 (0.83–0.92)	0.00
**CAP**	267	49	0.64 (0.58–0.71)	1.00	53	0.66 (0.59–0.73)	1.00	49	0.54 (0.47–0.61)	1.00	16	0.50 (0.41–0.59)	1.00
**PRO-C3**	250	44	0.63 (0.56–0.70)	1.00	48	0.64 (0.57–0.70)	1.00	45	0.60 (0.53–0.67)	1.00	13	0.69 (0.58–0.80)	0.97
**ALT**	335	47	0.64 (0.58–0.70)	1.00	50	0.65 (0.59–0.71)	1.00	47	0.52 (0.45–0.58)	1.00	15	0.51 (0.43–0.60)	1.00
**AST**	335	47	0.69 (0.63–0.75)	1.00	50	0.71 (0.65–0.76)	1.00	47	0.58 (0.52–0.65)	1.00	15	0.60 (0.52–0.68)	1.00
**CK18-M30**	239	42	0.74 (0.68–0.80)	0.97	47	0.75 (0.69–0.81)	0.94	42	0.63 (0.56–0.70)	1.00	13	0.64 (0.54–0.74)	1.00
**CK18-M65**	236	42	0.78 (0.72–0.84)	0.76	46	0.78 (0.72–0.84)	0.77	43	0.66 (0.59–0.73)	1.00	13	0.65 (0.55–0.74)	1.00
**FIB-4**	331	46	0.64 (0.58–0.70)	1.00	50	0.63 (0.57–0.69)	1.00	47	0.74 (0.69–0.80)	0.98	15	0.78 (0.72–0.84)	0.77
**ADAPT**	246	43	0.65 (0.58–0.72)	1.00	48	0.64 (0.57–0.71)	1.00	45	0.76 (0.71–0.82)	0.88	13	0.80 (0.72–0.89)	0.46
**ELF**	276	46	0.67 (0.60–0.73)	1.00	50	0.65 (0.59–0.72)	1.00	46	0.74 (0.68–0.80)	0.98	14	0.82 (0.75–0.89)	0.26
**NIS2**+	221	43	0.82 (0.77–0.88)	0.19	47	0.83 (0.77–0.88)	0.15	40	0.70 (0.63–0.77)	1.00	14	0.63 (0.53–0.73)	1.00
**FAST**	190	50	0.73 (0.66–0.80)	0.97	53	0.73 (0.66–0.80)	0.97	48	0.69 (0.61–0.76)	1.00	16	0.76 (0.67–0.85)	0.80
**Agile 3**+	288	48	0.68 (0.62–0.74)	1.00	52	0.65 (0.58–0.71)	1.00	47	0.84 (0.80–0.89)	0.03	14	0.89 (0.84–0.94)	0.00
**Agile 4**	288	48	0.66 (0.60–0.72)	1.00	52	0.63 (0.57–0.70)	1.00	47	0.82 (0.77–0.87)	0.18	14	0.88 (0.83––0.94)	0.00
**cTAG**	187	49	0.70 (0.63––0.78)	0.99	53	0.71 (0.64–0.79)	0.99	53	0.69 (0.61–0.77)	1.00	14	0.71 (0.59–0.82)	0.95
**MEFIB**	252	47	0.64 (0.59–0.70)	1.00	50	0.62 (0.57–0.68)	1.00	48	0.72 (0.67–0.77)	1.00	15	0.76 (0.69–0.84)	0.81
**MAST**	203	52	0.78 (0.71–0.84)	0.75	55	0.78 (0.71–0.84)	0.77	52	0.78 (0.72–0.85)	0.71	15	0.80 (0.72–0.87)	0.53

Diagnostic performance was assessed using AUCs that were estimated using receiver operating characteristic (ROC) analysis. The 95% CIs were calculated using the DeLong method. *P* values correspond to one-sided hypothesis tests of AUCs exceeding 0.80 (the predefined minimum acceptable criterion). Values were rounded to two decimal places. No adjustment for multiple comparisons was applied.

The highest AUCs for imaging, blood based and composite scores for the diagnosis of at-risk MASH were, respectively, 0.75 (0.69–0.81) for MRE, 0.82 (0.77–0.88) for NIS2+ and 0.78 (0.71–0.84) for MRI-AST score (MAST). Of the top 3 performing markers, 2 were blood based (NIS2+ as above and cytokeratin 18 measured using the M65 assay (CK18-M65) (AUC 0.78 (0.72–0.84)) and one was a composite score (MAST, AUC 0.78 (0.71–0.84)). Other magnetic resonance imaging (MRI) markers had AUCs below 0.7 (proton density fat fraction (PDFF) measured using Liver MultiScan (Perspectum; LMS-PDFF) and LMS (Perspectum) for iron-corrected T1 (LMS-cT1), both AUC 0.66 (0.59–0.72); vendor-PDFF, 0.59 (0.51–0.68); and MRI-NASH index measured using ‘detection of metabolic liver injury’ MRI (deMILI MRI-NASH), 0.62 (0.53–0.71)). The AUC for MR-based MAST (AUC 0.78 (0.71–0.84)) was numerically higher than that for the VCTE-LSM-based Fibroscan-AST (FAST; 0.73, 0.66–0.80).

**Extended Data Fig. 8:**
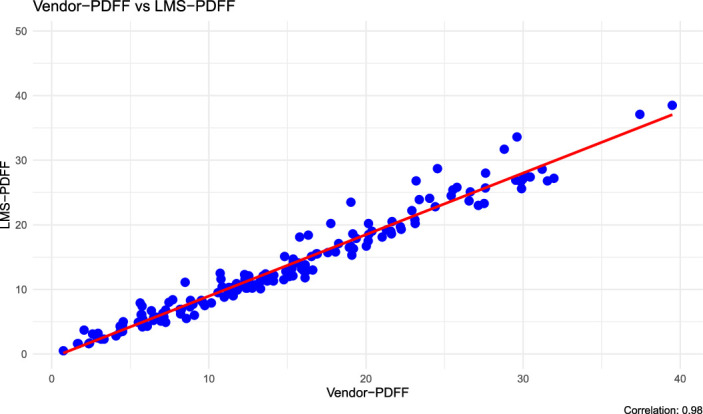
Association between proton density fat fraction (PDFF) measured using. Liver Multiscan (LMS-PDFF) and PDFF measured using the MR vendor specific sequences (vendor-PDFF). There was a very high correlation (r = 0.98) between LMS-PDFF and vendor-PDFF.

For MASH, the highest AUCs for imaging, blood based and composite scores were, respectively, 0.71 (0.65–0.77) for MRE, 0.83 (0.77–0.88) for NIS2+ and 0.78 (0.71–0.84) for MAST. Of the top 3 performing markers, 2 were blood based (NIS2+ as above and CK18-M65, AUC 0.78 (0.72–0.84)) and one was a composite score (MAST, AUC 0.78 (0.71–0.84)). Composite scores performed better than imaging alone, with MAST, 0.78 (0.71–0.84), and FAST, 0.73 (0.66–0.80). LMS-PDFF (AUC 0.70, 95% confidence interval (CI) 0.64–0.76) had a numerically higher AUC than the controlled attenuation parameter (CAP; 0.66; 95% CI 0.59–0.73), LMS-cT1 (0.68; 95% CI 0.61–0.74) and vendor-PDFF (0.67; 95% CI 0.58–0.75) for the diagnosis of MASH. LMS-PDFF and vendor-PDFF were highly correlated (Extended Data Fig. 8). In head-to-head analysis of cases in which measurements of both LMS-PDFF and vendor-PDFF were available, LMS-PDFF had a numerically higher AUC than vendor-PDFF for at-risk MASH and MASH, and vendor-PDFF had a numerically higher AUC for fibrosis indications (Supplementary Table 2).

### Diagnostic performance for advanced fibrosis and cirrhosis

**Fig. 2:**
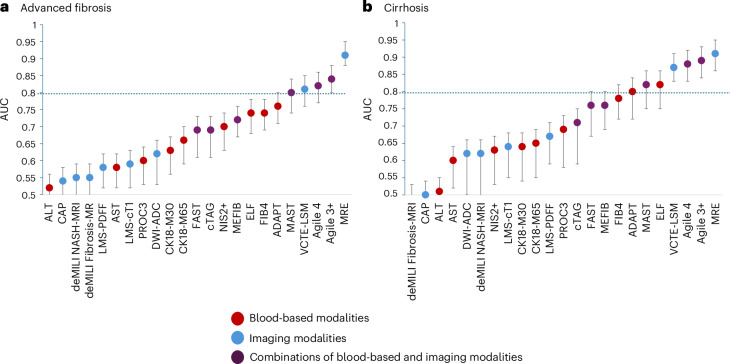
Diagnostic performance of biomarkers for advanced fibrosis and cirrhosis. Each point in this figure represents the AUC estimate for a marker, and error bars indicate 95% CIs for AUC. Biomarkers are grouped by modality (blood-based, imaging and combination markers). The unit of analysis was the individual participant. AUC estimates and corresponding 95% CIs were derived from patient-level data. A dashed line to indicate the a priori-defined MAC of the AUC of 0.80 is included in each graph. Sample sizes varied across biomarkers as follows: MRE (*n* = 275), LMS-cT1 (*n* = 265), LMS-PDFF (*n* = 304), vendor-PDFF (*n* = 163), deMILI fibrosis-MRI (*n* = 158), deMILI NASH-MRI (*n* = 158), DWI-ADC (*n* = 160), VCTE-LSM (*n* = 298), CAP (*n* = 267), PRO-C3 (*n* = 250), ALT (*n* = 335), AST (*n* = 335), CK18-M30 (*n* = 239), CK18-M65 (*n* = 236), FIB-4 (*n* = 331), ADAPT (*n* = 246), ELF (*n* = 276), NIS2+ (*n* = 221), FAST (*n* = 190), Agile 3+ (*n* = 288), Agile 4 (*n* = 288), cTAG (*n* = 187) and MAST (*n* = 203). **a**, Advanced fibrosis: the highest AUCs for blood-based, imaging and combination markers were, respectively, 0.76 (0.71–0.82) for ADAPT, 0.91 (0.88–0.95) for MRE and 0.91 (0.88–0.95) for Agile 3+. Both MRE and Agile 3+ met the MAC. **b**, Cirrhosis: the highest AUCs for blood-based, imaging and combination markers were, respectively, 0.82 (0.75–0.89) for ELF, 0.91 (0.86–0.95) for MRE and 0.89 (0.84–0.94) for Agile 3+. MRE, Agile 3+, Agile 4 (AUC 0.88 (0.83–0.94)) and VCTE-LSM (AUC 0.87 (0.83–0.92)) met the MAC.

The diagnostic performance of the biomarkers for advanced fibrosis (F≥3) or cirrhosis (F4) ranged from an AUC of 0.49 to 0.91. For fibrosis endpoints, elastography (imaging) biomarkers tended to outperform serum biomarkers (Fig. 2).

For advanced fibrosis, the highest AUCs for imaging, blood based and composite scores were, respectively, 0.91 (0.88–0.95) for MRE, 0.76 (0.71–0.82) for ADAPT (a multi-marker score that includes the parameters of age, presence of diabetes, platelet count and serum level of Pro C3) and 0.84 (0.80–0.89) for Agile 3+ (a composite score that includes the parameters of LSM, AST, ALT, age, platelet count, presence of type 2 diabetes and sex). The three highest-performing biomarkers were imaging markers (MRE as above) or composite scores that included an elastography measurement (Agile 3+ as above and Agile 4 (AUC 0.82 (0.77–0.87)). MRE and Agile 3+ exceeded the MAC for advanced fibrosis. Non-elastography MR markers performed less well (LMS-cT1: 0.59 (0.52–0.66); MRI-fibrosis index measured using deMILI MRI (deMILI fibrosis-MRI): 0.55 (0.46–0.64); diffusion-weighted imaging-derived apparent diffusion coefficient (DWI-ADC): 0.62 (0.53–0.71)). Other blood-based biomarkers reached only modest performance similar to ADAPT (fibrosis-4 index (FIB-4): AUC 0.74 (0.69–0.80); enhanced liver fibrosis test (ELF): AUC 0.74 (0.68–0.80)).

For cirrhosis, the highest AUC for imaging, blood based and composite scores were, respectively, 0.91 (0.86–0.95) for MRE, 0.82 (0.75–0.89) for ELF and 0.89 (0.84–0.94) for Agile 3+. The top three performing markers were imaging markers (MRE as above) and composite scores (Agile 3+ as above and Agile 4, AUC 0.88 (0.83–0.94)). MRE, Agile 3+, Agile 4 and VCTE-LSM (AUC 0.87 (0.83–0.92)) all exceeded the MAC for cirrhosis. Non-elastography MR markers performed less well (LMS-cT1: 0.64 (0.55–0.73); deMILI fibrosis-MRI: 0.49 (0.37–0.61); DWI-ADC: 0.62 (0.50–0.73)).

The results of the sensitivity analysis using standardization, making the subgroups of available results more similar with respect to common confounders, were not fundamentally different from those of the main analysis (Supplementary Tables 3 and 4).

### Head-to-head comparisons

**Fig. 3:**
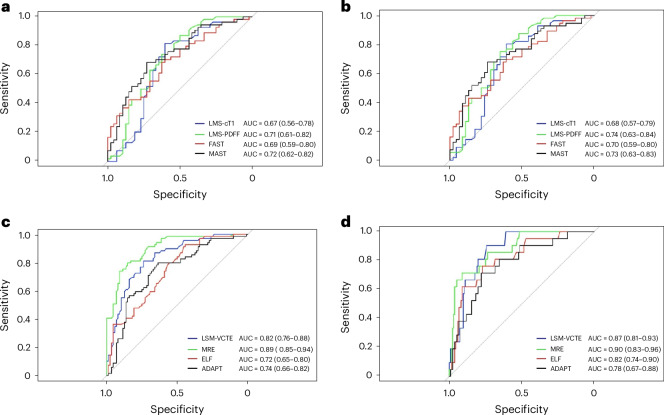
Head-to-head comparison of selected biomarkers. Area under the receiver operating characteristic curves for selected imaging and composite biomarkers for each of the four target conditions in our study including cases in which all biomarkers were available for analysis. **a**, At-risk MASH: (FAST, LMS-PDFF, LMS-cT1, MAST), *n* = 102. **b**, MASH: (FAST, LMS-PDFF, LMS-cT1, MAST), *n* = 102. **c**, Advanced fibrosis (F3+): (VCTE-LSM, MRE, ELF, ADAPT), *n* = 157. **d**, Cirrhosis (F4): (VCTE-LSM, MRE, ELF, ADAPT), *n* = 157.

Direct head-to-head comparisons were carried out for preselected biomarkers with sufficient data. The performance of LMS-cT1, LMS-PDFF, FAST and MAST were tested in 102 of 357 participants (29% of the analysis set) in which all four biomarkers were available. There was considerable overlap in the confidence interval of the AUC both for the evaluation of at-risk MASH (Fig. 3a) and MASH (Fig. 3b). In the 157 of 357 (44% of the analysis set) participants with available fibrosis biomarkers, MRE was superior to ELF and ADAPT for the assessment of advanced fibrosis, but the confidence intervals of the AUC for MRE and VCTE-LSM overlapped (Fig. 3c). For cirrhosis, MRE was superior to ADAPT but confidence intervals overlapped between all other biomarkers (Fig. 3d). The characteristics of the participants that were included in the head-to-head comparisons are shown in Supplementary Table 5.

### Performance of biomarkers at prespecified thresholds

The diagnostic performance of a number of biomarkers at previously specified thresholds was tested for at-risk MASH and advanced fibrosis. For at-risk MASH, high sensitivity was achieved at the expense of low specificity and vice versa (Table 3). For advanced fibrosis, MRE at a threshold of 3.5 kPa had sensitivity of 0.71 and specificity of 0.92, and VCTE-LSM at a threshold of 10 kPa had sensitivity of 0.73 and specificity of 0.74 (Table 3). A comparison of the diagnostic performance achieved in this study with the performance reported in the literature is included in Supplementary Tables 6 and 7.

**Table 3:** Performance of markers at predefined thresholds

Biomarker	*n*	Threshold	Sensitivity	Specificity
**Biomarker performance for at-risk MASH**				
**MRE (kPa)**	275	3.3	0.69 (0.56–0.73)	0.76 (0.68–0.82)
**LMS-cT1 (ms)**	265	875	0.58 (0.49–0.66)	0.61 (0.53–0.69)
**LMS-PDFF (%)**	304	8	0.89 (0.84–0.94)	0.32 (0.25–0.40)
**Vendor-PDFF (%)**	163	8	0.86 (0.18–0.94)	0.28 (0.19–0.73)
**deMILI MRI-NASH (0–1)**	158	0.5	0.58 (0.47–0.70)	0.60 (0.49–0.70)
**CAP (dB** **m**^**−1**^**)**	267	280	0.92 (0.87–0.97)	0.32 (0.25–0.41)
**NIS2**+	221	0.68	0.72 (0.62–0.81)	0.81 (0.74–0.88)
**FAST**	190	0.67	0.41 (0.31–0.51)	0.86 (0.79–0.93)
**cTAG (%)**	187	0.34	0.55 (0.45–0.66)	0.76 (0.67–0.84)
**MAST**	203	0.242	0.41 (0.32–0.51)	0.91 (0.84–0.96)
**Biomarker performance for advanced fibrosis**				
**MRE (kPa)**	275	3.5	0.71 (0.63–0.79)	0.92 (0.87–0.96)
**deMILI MRI-fibrosis (0–1)**	158	0.5	0.18 (0.10–0.89)	0.76 (0.20–0.85)
**VCTE-LSM (kPa)**	298	10	0.73 (0.66–0.81)	0.74 (0.67–0.81)
**Agile 3**+	288	0.679	0.65 (0.57–0.73)	0.84 (0.78–0.90)
**Agile 4**	288	0.565	0.20 (0.13–0.26)	0.99 (0.97–1.00)
**MEFIB**	252	MRE ≥ 3.3 and FIB-4 ≥ 1.68^a^	0.51 (0.42–0.60)	0.93 (0.89–0.97)

^a^The specific criterion used to dichotomize the test.

## Discussion

In this diagnostic accuracy study of multiple imaging and serum biomarkers, performance was assessed for the diagnosis of four histologically defined target conditions addressing disease activity (MASH; at-risk MASH: MAS ≥ 4 with *F* ≥ 2) and staging of hepatic fibrosis (advanced fibrosis and cirrhosis). The population under evaluation were people with MASLD who were already assessed in secondary and tertiary care centers and were referred for biopsy for disease evaluation; our findings should not be extrapolated to other settings such as screening in primary care and in type 2 diabetes. We would, however, highlight that, while the pretest probability may differ between primary care and secondary–tertiary care populations, the sensitivity, specificity and area under the receiver operating characteristic curve (AUROC) data presented in this paper remain relevant.

The scale of the study in its geographical distribution, number of recruiting centers, size of the recruited cohort, central histology evaluation and scope of biomarkers assessed (both the range of MR and ultrasound modalities alongside numerous blood-based biomarkers) is unique. These key strengths led to the minimization of potential bias, in contrast to previous studies, and therefore, our results represent a truly robust external validation for biomarker performance.

For detection of MASH or at-risk MASH, none of the evaluated biomarkers significantly exceeded the prespecified MAC. However, blood-based biomarkers tended to perform better than imaging for activity-related targets. Considering advanced fibrosis and cirrhosis, elastography techniques (MRE and VCTE-LSM) and composite scores that include liver stiffness measurements (Agile 3+, Agile 4) showed the best performance, with MRE and Agile 3+ significantly exceeding the MAC for both advanced fibrosis and cirrhosis, and VCTE-LSM and Agile 4 significantly exceeding the MAC for cirrhosis.

Patients with at-risk MASH are the key target group for prescription of pharmacotherapy for pre-cirrhotic MASH^15^, as well as for entry into clinical trials. Overall, similar performance characteristics were found for the at-risk MASH and the MASH target conditions (Table 2). The highest AUC for at-risk MASH was achieved by NIS2+ (0.82, 0.77–0.88), a result comparable to that reported in previous studies assessing this biomarker (0.81, 0.79–0.83)^16^. Among single imaging markers, elastography techniques had the highest AUC for at-risk MASH (MRE: 0.75; VCTE-LSM: 0.71). This suggests that the fibrosis component of at-risk MASH classification has a strong influence, and provides the rationale for including stiffness in composite scores such as FAST and MAST.

For at-risk MASH, other MR imaging biomarkers achieved numerically lower AUCs: LMS-cT1 and LMS-PDFF, both with an AUC of 0.66 (0.59–0.72). Previous studies that examined these two markers reported similar performances. A study from Japan reported an AUC of 0.74 for LMS-cT1 and 0.71 for LMS-PDFF^17^, while a pooled multicenter study from the UK reported an AUC of 0.78 for LMS-cT1 and 0.69 for LMS-PDFF^18^.

In this study, cTAG (a composite score that includes the parameters of cT1, AST and fasting serum glucose level) had an AUC of 0.70 (0.63–0.78), compared with an AUC of 0.90 when first described^19^, while deMILI NASH-MRI had an AUC of 0.62 (0.53–0.71) compared with a previously reported AUC of 0.86 (ref. ^20^). The study that described cTAG was a retrospective analysis of a prospectively recruited pooled cohort, while the study that described the deMILI NASH-MRI score was a small single-center study. These factors, and the lack of central histological evaluation in the published studies, are likely to have introduced bias that led to overestimation of the diagnostic performance.

The performance of routine clinical biochemistry markers such as AST (AUC 0.69, 0.63–0.75) can provide a useful barometer for the incremental value of other tests. deMILI NASH-MRI (AUC 0.62) and MEFIB (an index that uses data from MRE and FIB4) (0.64) achieved a lower AUC than AST while a numerically higher AUC was observed in cTAG (0.70), VCTE-LSM (0.71), FAST (0.73), MRE (0.75), MAST (0.78), CK18-M65 (0.78) and NIS2+ (0.82). These are indicative observations with overlapping 95% CIs for the AUC in the overall and head-to-head analyses so superiority could not be firmly established.

The performance of biomarkers for the diagnosis of ‘at-risk MASH’ in the country-adjusted analysis (Supplementary Table 4) presents a very similar picture to the unadjusted analysis. MRE liver stiffness remains numerically the highest-performing imaging biomarker albeit with a lower AUC of 0.69 (0.61–0.75). Performance of NIS2+ remains stable, and this biomarker remains best performing albeit below the MAC.

The performance of composite scores in our study was, in general, lower than previously described. A previous metanalysis reported a summary area under the receiver operating characterostic curve of 0.79 (0.77–0.81) for FAST^21^ (compared with AUC of 0.73 in this study). The index studies in that meta-analysis were in many cases retrospective and included a variety of populations (for example, screening population in the AURORA clinical trial^22^, bariatric surgery cohorts^23^). These factors are likely to have introduced bias that inflated the previously reported performance of FAST. MAST was previously evaluated in small studies from one or two centers that reported AUCs of 0.80 (0.72–0.89)^24^ and 0.93 (0.88–0.97)^25^, respectively (compared with AUC of 0.78 in this study). In a cohort with type 2 diabetes, FAST (AUC 0.81) and MAST (AUC 0.79) were also reported to have higher performance for at-risk MASH^26^, but it is difficult to compare these results with those of our study owing to differences in the population being evaluated. Compared with previous studies that are subject to possible bias from study design, heterogeneity of the included population and lack of multicenter prospective validation, our study provides robust external validation for biomarker performance that is closer to the true biomarker performance.

Our data suggest that measures of disease activity that comprise biologically plausible circulating markers of inflammation are promising for use in detecting MASH. It was therefore surprising that despite our methodological rigor none of the biomarkers met the MAC for MASH and at-risk MASH. We believe that this reflects inherent bias in the current histological definition of MASH. It is well established that the histological features of ballooning and lobular inflammation are subject to substantial observer-dependent variability^9^. Furthermore, disease activity is known to be heterogenous across the liver, leading to biopsy sampling error, and can also fluctuate with time. These factors may contribute to variation in the approximation of histology to the true status of the liver as a whole, but as histology is the reference standard, the performance of the blood-based or imaging biomarker would still be penalized, even if it were a more faithful approximation in reality.

Turning to the evaluation of fibrosis, elastography techniques (MRE and VCTE-LSM) and composite scores that include liver stiffness measurements (Agile 3+, Agile 4) had the best performance. MRE performed best overall for advanced fibrosis (0.91, 0.88–0.95) and cirrhosis (0.91, 0.86–0.95). These results were similar to the findings of two previous meta-analyses, which reported summary AUCs 0.92 (0.88–0.95)^27^ and 0.92 (0.90–0.94)^28^ for advanced fibrosis and 0.90 (0.81–0.95)^27^ and 0.94 (0.92–0.96)^28^ for cirrhosis. The next best performing markers were Agile 3+ and Agile 4, while VCTE-LSM as a stand-alone measure showed AUCs of 0.81 (0.76–0.86) and 0.87 (0.83–0.92) for advanced fibrosis and cirrhosis, respectively, which again aligned with the findings of previous meta-analyses^27,29^. Among the blood-based markers and multi-marker scores, ELF and the PRO-C3-based ADAPT score had AUCs ranging from 0.74 to 0.76 and 0.80 to 0.82 for advanced fibrosis and cirrhosis, respectively. These results are similar to those observed in the separate LITMUS Metacohort study and the NIMBLE study^30,31^.

The diagnostic performance of biomarkers at prespecified thresholds was generally lower in our analysis compared with previous reports (Supplementary Tables 6 and 7). This finding is most probably due to the prospective nature of our study, which would be less prone to bias. By contrast, some of the studies in the literature were small^19,25^ or used multiple-participant groups in meta-analyses^18,29,32^, something that may have introduced bias and overestimation of performance.

Our analysis relating to biomarker performance at predefined thresholds produced some results worth further commentary. For at-risk MASH, some biomarkers have complementary performance and could potentially be combined in two-step approaches. For example, tests with high sensitivity (such as PDFF at a threshold of 8% (sensitivity 0.89) or CAP at a threshold of 280 dB m^−1^ (sensitivity 0.92)) can be applied first, followed by tests with high specificity such as MRE at 3.3 kPa (sensitivity 0.76) or FAST at a threshold of 0.67 (specificity 0.86) or MAST at a threshold 0.242 (specificity 0.91). Such approaches could be used in screening for clinical trial recruitment to help reduce screen failure rates. With increasing numbers of approved pharmacotherapies, such approaches can also help with selecting patients suitable for treatment.

The results for the performance of biomarkers at prespecified thresholds for advanced fibrosis reflect the results of the AUC analysis and confirm that elastography techniques and composites scores that include elastography achieve high sensitivity and specificity.

Major strengths of this study include truly international, multicenter, prospective recruitment and the study being specifically designed to robustly assess biomarker performance. Unlike previous imaging studies, which tend to be single-center cohorts recruited based on convenience and focusing on single modalities, the LITMUS Imaging Study is the product of a major international collaboration across 13 countries in Europe and North America^33,34^.

**Extended Data Fig. 9:**
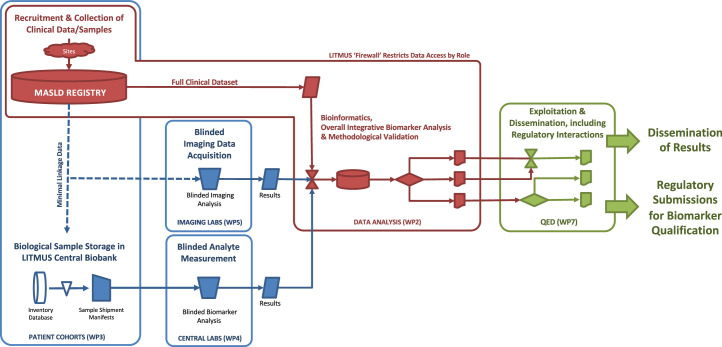
The LITMUS firewall ensured blinded data flows between recruitment sites, central analysis labs and statistical analysis. The current study was underpinned by rigorous quality control procedures that ensured a tightly defined chain-of-custody for all data and biological samples. Liver biopsy slides were centrally processed and read by internationally recognised expert hepato-pathologists from the LITMUS Histopathology Group. MR biomarker data were processed centrally and analysed in imaging core labs specialised in the relevant technologies. Similarly, all biological sample handling followed strict standard operating procedures to minimise pre-analytical variation with circulating biomarker analysis conducted in a centralized CLIA-certified laboratory. The technicians conducting all analyses were blinded to associated clinical data including the results of other biomarkers and liver histology.

This study was underpinned by rigorous quality control procedures that ensured a tightly defined chain of custody for all data and biological samples (Extended Data Fig. 9)^33^. Liver biopsy slides were centrally processed and read by internationally recognized expert hepatopathologists from the LITMUS Histopathology Group. MR biomarker data were processed centrally and analyzed in imaging core laboratories specialized in the relevant technologies. Similarly, all biological sample handling followed strict standard operating procedures to minimize pre-analytical variation with circulating biomarker analysis conducted in a centralized Clinical Laboratory Improvement Amendments (CLIA)-accredited laboratory. The technical personnel conducting all analyses were blinded to associated clinical data including the results of other biomarkers and liver histology. Ultimately, the diverse datasets generated across the study were assembled for statistical analysis led by an independent statistician with specific methodological expertise in the assessment of biomarker performance and no imperative to show superior performance of any test over the others.

Limitations also need to be acknowledged, in particular the limited sample size due to the study costs, especially when head-to-head comparisons were considered. However, it should be noted that outside of clinical trials, in which cases are heavily prescreened, this is a relatively large prospectively recruited study designed with the specific aim of assessing biomarker diagnostic accuracy and with centrally read histology as the reference standard. Recruitment was conducted in specialist secondary and tertiary care centers, and so, factors such as differences in prevalence, epidemiology, referral patterns and clinical workup before biopsy may affect the generalizability of our findings to other settings. As described above, histology is an imperfect reference standard being subject to sampling variation as well as both inter- and intraobserver variation in reading^9^. With a theorized 90% sensitivity and 90% specificity for biopsy itself, it has been reported that the highest performance a comparative marker could reasonably achieve in a 40% prevalence setting is an AUC of 0.90 (ref. ^35^). Despite the limitations of a histological reference standard, this study identified biomarkers that achieved and approached that theoretical threshold with statistical significance for fibrosis indications. Lastly, our cohort is predominantly made up of people of white ethnicity (94%). Our results may therefore not be representative of biomarker performance in other ethnic groups or diverse populations.

It should also be noted that the definition of the target condition of at-risk MASH in this study included those with MASH and F2–4 fibrosis. This definition was used when the term was first described and is included in the majority of the literature^16,18,32^. In clinical practice, there is an overriding need to differentiate between those with a degree of MASL associated with cardio-metabolic risk factors and those with progressive liver disease that are at greatest risk of liver-related adverse outcomes (true at-risk MASH), so this definition is most relevant. However, there is also value in distinguishing cases with cirrhosis from those with pre-cirrhotic MASH. While not addressed in our study, other studies have examined the performance of biomarkers for MASH + F2–3 either using single cutoffs^36^ or upper and lower cutoffs^37^.

Our study has important implications for clinical practice indicating that MRE and Agile 3+ can be used to positively rule-in advanced fibrosis and cirrhosis, while VCTE-LSM and Agile 4 can be used to rule-in cirrhosis. Therefore, these biomarkers can be used as confirmatory tests for disease severity once patients are referred to secondary or tertiary care. Biomarkers that did not meet the MAC for fibrosis assessment should remain investigational and should be examined in different contexts (for example, screening in primary care or in diabetes clinics). The fact that none of the biomarkers met the MAC for MASH indications raises serious concerns about the validity of MASH as a target condition. The use of MASH as part of endpoint definitions in clinical trials and as a potential feature to be used when selecting patients for treatment with approved pharmacotherapies should therefore be re-examined in view of our results.

While our study provides useful data on biomarker performance, we are limited by the use of histological target conditions. Establishing the performance of biomarkers for the prognosis of adverse outcomes would be more clinically relevant. The longitudinal data being generated within the European Steatotic Liver Disease (MASLD) Registry will be an important asset for assessing the prognostic value of biomarkers and addressing this evidence gap. Furthermore, future studies are also needed to address the performance of biomarkers in other contexts such as monitoring or assessing therapeutic response. Lastly, future health economics studies should evaluate whether the superior performance of the more expensive tests such as MR biomarkers and proprietary serum-based tests translates into cost-effectiveness.

The results of our study rigorously show the accuracy of both staple imaging tests and blood-based biomarkers proposed for use in MASLD. While highly performant biomarkers to assess MASH and at-risk MASH remain elusive, with none achieving the MAC, we have robustly characterized the performance of multiple potentially tractable biomarkers. Noninvasive assessment of fibrosis was more accurate, with elastography technologies performing best. It should, however, be noted that circulating direct collagen biomarkers such as ELF and PRO-C3 may offer additional insights into disease biology, such as the balance of fibrogenesis and fibrolysis, which provides complementary information beyond the measurement of stable hepatic fibrosis.

To date, no biomarkers have been approved by the FDA or European Medicines Agency as reasonably likely surrogate endpoints for MASLD drug development. Our findings serve as an important addition to the literature in support of advancing biomarkers toward regulatory qualification. These data will also inform clinical guideline development, supporting clinicians as they select the most tractable tests to improve the clinical care and outcomes of patients with MASLD.

## Methods

### Study design and recruitment

This LITMUS Imaging Study (NCT05479721, clinicaltrials.gov) was a prospective study of diagnostic accuracy, conducted as part of the EU IMI-2 LITMUS research program, to evaluate the performance of multiple noninvasive biomarkers for the diagnostic context of use^34^. Participants with suspected MASLD referred for investigation at liver centers across 13 countries in Europe and the USA between January 2018 and June 2022 were prospectively recruited into the European Steatotic Liver Disease Registry ‘LITMUS Study Cohort’^33^. Those participants recruited at centers where the requisite MR imaging capabilities were available were invited to participate in the nested LITMUS Imaging Study^34^. Clinical data, imaging acquisition and biological samples were collected within 6 months of diagnostic liver biopsy.

Participants were reimbursed for travel and food expenses relating to their appointments for MR assessments. These appointments were not part of the routine clinical care, and participants were asked to attend after at least a 4-h fast.

### Ethical oversight

The study was conducted in line with the ethical principles for medical research involving human study participants, as specified in the Declaration of Helsinki. The project was approved by the relevant ethical committees in the participating countries, and all participants provided written informed consent before inclusion. In the UK, the LITMUS Imaging Study was approved by the London Queen Square Research Ethics Committee (18/LO/1953); in France by Comite de protection des personnes, Sud Mediterranee IV (reference CPP: 19 06 04; ID-RCB:2019-A01308-49) for Paris, and Comite de Protection des Personnes (CPP) Ouest II, Angers (CPP number CB-2010-01, dossier number DC-2011-1467, dossier number AC 2014-2329) for Angers; in Spain by CEI de los Hospitales Universitarios Virgen Macarena y Virgen del Rocío (Informe Dictamen Favorable, Proyecto Investigación Biomédica, C.P. LITMUS_GA_777377 - C.I, 20 de Septiembre de 2018) for Seville, Drug Research Ethics (Vall d’Hebron) for Barcelona (PR(AG)507/2020) and CEIm Area de Salud Valladolid Este, University Hospital Valladolid (18-1173) for Valladolid; in the USA, initial approval was given by Integ Review IRB (protocol number LI-001-2019) and continued approval by ADVARRA IRB (continuing review approval CR00408748); in Italy by A.O.U. City of Health and Science of Turin—A.O. Mauritian Order—ASL TO 1 (Prot N. N 0125391 18 Dec 2018) for Torino, and Comitato Etico Palermo 1, Azienda Ospedaiera Universitaria Policliniclo Paolo Giaccone di Palermo (Verbale N. 11/2019, 16.12.2019) for Palermo; in Germany by Ethikkommission der Landesärztekammer Rheinland-Pfalz (2018-13269_1, 19.08/2018); in Sweden by Regionala etikpövningsnämnden I Linköping (Dnr 2018/393-31); in Switzerland by Kantonale Ethikkommission (KEK), Bern (KEK Nr 2019-00609); and in Finland by HUS eettinen toimikunta IV (HUS/1784/2019).

### Patient selection

Adults (aged ≥18 years) of male and female sex undergoing a liver biopsy performed as part of their standard diagnostic assessment for presumed MASLD with paired imaging markers were consecutively included. Patients with excessive alcohol intake (>20–30 g d^−1^) or evidence of other chronic liver conditions, such as viral hepatitis B or C, were excluded from the study. The sex of each participant was determined based on self-reported information.

### Histological assessment

Biopsy samples were evaluated centrally by liver pathologists from the LITMUS Histopathology Group (LHG), a team of ten expert hepatopathologists who were blinded to clinical data and had previously harmonized in scoring MASLD features with high interobserver agreement^38^. Each sample was consensus scored by two pathologists, with any discrepancies being adjudicated by the LHG chair.

MASLD activity was assessed using the MASH Clinical Research Network scoring system. Steatosis and lobular inflammation were rated on four-point scales (0–3) and hepatocyte ballooning on a three-point scale (0–2). The MAS, calculated as the sum of steatosis, inflammation and ballooning scores, ranges from 0 to 8. The staging of liver fibrosis was performed on a five-point scale (F0–F4)^3^.

A liver biopsy with no more than 3 fragments, at least 1.5 cm in length and containing at least 6 portal tracts was considered adequate for the diagnosis of MASLD and MASH by the LHG. In very few cases that did not meet the quality recommendations above, if all histological features for diagnosing MASH, according to the accepted minimum histological criteria, were clearly seen in a biopsy specimen despite suboptimal quality, the specimen was considered adequate. The above is in keeping with the consensus for the standardized application of histological grading and staging systems in MASH by the International MASLD Pathology Group^39^, whose members include the majority of the LHG. Biopsies that did not meet the set adequacy criteria and did not show the minimal histological features for diagnosing MASH were rejected.

### Clinical assessment

In each center, a trained investigator collected detailed clinical data on all participants (including sex assigned at birth) and entered them directly into the web-based registry portal. BMI was calculated by dividing weight (kg) by height (m) squared. Clinical laboratory tests, including platelet count, LDL, HDL, cholesterol, triglycerides and GGT, were performed in laboratories of the respective recruitment centers and recorded in the registry.

Common comorbidities were also documented in each center, including dyslipidemia (fasting triglyceride level ≥ 150 mg dl^−1^ (1.7 mmol l^−1^) or fasting HDL < 40 mg dl^−1^ (1.03 mmol l^−1^) in males and <50 mg dl^−1^ (1.29 mmol l^−1^) in females; or on treatment), hypertension (systolic blood pressure ≥ 130 mm Hg or diastolic blood pressure ≥ 85 mm Hg) and diabetes (raised fasting glucose ≥ 100 mg dl^−1^ (5.6 mmol l^−1^), HbA1c ≥ 48 mmol mol^−1^ (6.5%) or previously diagnosed insulin resistance or type 2 diabetes mellitus).

### Biomarkers

To ensure robustness of the analysis, all imaging data processing and biological sample analyses were conducted by trained operators at central laboratories blinded to all associated clinical data including the results of the liver biopsies and all other biomarker data (Supplementary Table 8 and Extended Data Fig. 9).

### Imaging

Liver stiffness measurement by VCTE-LSM and ultrasound attenuation (CAP) were measured using FibroScan (Echosens) devices at the sites as per the manufacturer’s recommendations^40^. Six quantitative MRI markers were measured on MRI systems manufactured by Siemens, GE and Philips, at either 3 T or 1.5 T field strength, depending on availability at each site. These included LMS-cT1 (based on the combination of T1 and T2*)^41^ and MRE, which measures liver stiffness (Resoundant; technology licensed to major MRI vendors)^42^. DWI-ADC^43^ and two radiomics-based metrics: Fibro MRI and NASH-MRI measured using deMILI were also evaluated^20^. Proton density fat fraction was measured using both Liver Multiscan (LMS-PDFF)^44^ and in some cases PDFF sequences specific to each MR scanner vendor (vendor-PDFF). Technical details for all the imaging procedures are available in full in the study protocol^34^, and for ease of reference, some key aspects of the MR procedures are also summarized in Supplementary Methods.

### Blood-based markers

Serum samples were collected using standardized kits and stored at −80 °C according to the LITMUS prespecified biobanking procedures^33^. Samples were shipped in batches on dry ice from recruitment sites to the Integrated Biobank of Luxembourg (IBBL) that was used as the LITMUS Central Biobank, cataloged and then sent to the LITMUS Central Laboratory at Nordic Biosciences, a laboratory accredited by the College of American Pathologists. Due to differences in available sample volumes, not all biomarkers were measured for every participant.

The following biomarkers were measured at the LITMUS Central Laboratory (see Supplementary Table 8 for details): ALT, AST, CK18-M30 (M30 Apoptosense ELISA 10011, VLVbio)^45^, CK18-M65 (M65 EpiDeath ELISA 10040, VLVbio)^45^, Nordic-PRO-C3 (ELISA based)^46^, hyaluronic acid, TIMP-1 and PIIINP for ELF (Siemens)^47^.

Multi-marker scores were defined as scores that include data from multiple blood-based markers. The following multi-marker scores were calculated using their originally published formulae: Fibrosis-4 (FIB-4)^48^, ADAPT^49,50^, NIS2+ (refs. ^16,36^) and ELF. The NIS2+ scores were calculated at the Genfit Laboratory.

### Composite scores

Composite scores were defined as scores that are derived using data from blood-based and imaging markers. The following composite scores were calculated: FAST^32^, Agile 3+ (ref. ^51^), Agile 4 (ref. ^51^), MEFIB^52^, cTAG^19^ and MAST^25^ (see Supplementary Table 8 for details). FAST was computed solely when the time interval between the FibroScan procedure and the AST blood collection was 28 days or less.

### Target conditions

The diagnostic performance of the biomarkers was assessed for the detection of four clinically relevant target conditions.

#### Target condition 1: at-risk MASH

This target condition is characterized by clinically significant hepatic fibrosis (≥F2) with active steatohepatitis, defined as the presence of steatosis, lobular inflammation and hepatocellular ballooning with a MAS ≥4, with at least one point in each component. This target condition is stipulated by regulatory agencies including the FDA and the European Medicines Agency to define the key inclusion criterion for phase 2 and phase 3 clinical trials for treatment of pre-cirrhotic MASH.

#### Target condition 2: MASH

This target condition is characterized by active steatohepatitis, defined as the presence of steatosis, lobular inflammation and hepatocellular ballooning with a MAS score ≥4, with at least one point in each component, irrespective of the stage of fibrosis present.

#### Target condition 3: advanced fibrosis

This target condition is characterized by histological fibrosis stage ≥F3, irrespective of the grade of steatohepatitis activity.

#### Target condition 4: cirrhosis

This target condition is characterized by histological fibrosis stage F4, irrespective of the grade of steatohepatitis activity.

### Statistical analysis

#### Study group

The analysis was restricted to participants with data from at least one imaging modality (LITMUS Imaging Study group). Only results from imaging and blood samples collected within 6 months of a liver biopsy were included.

#### Diagnostic accuracy

We visualized the distribution of biomarker results across fibrosis stages and MAS as boxplots. Nonparametric, empirical ROC curves were constructed for each biomarker and multi-marker score for the respective target conditions. We then evaluated the ability of the biomarkers to replace liver biopsy by calculating the AUC and its 95% CI (DeLong)^53^. The AUC summarizes test performance across all possible thresholds, rather than at arbitrary cut‑offs, enabling fairer evaluation and comparison of multiple biomarkers. An AUC of 0.80 was established a priori as the MAC. One-sided hypothesis tests were used to evaluate whether the AUC exceeded this threshold, with *P* values below 0.05 indicating that the biomarker met the predefined performance criteria.

#### Standardization

Not all biomarker results were available for every participant. To make results comparable across biomarker subgroups, the probability of each participant being included in a specific subgroup was estimated using logistic regression, accounting for age, sex, BMI, type 2 diabetes and fibrosis stage. Propensity distributions were checked to avoid extreme values. A weighted ROC analysis with inverse propensity weighting was applied to standardize the subgroups and ensure representativeness. These analyses, identical to those of the main analysis described earlier, were performed for each target condition, using 999 bootstrap samples to calculate 95% CIs.

As the proportion of patients with at-risk MASH may differ between countries and subtle variations in marker values might occur owing to unaccounted ethnic or other extraneous differences between territories, both in those with and those without the target condition, unadjusted AUC estimates could hypothetically be slightly affected. To check for this potential source of bias, we also estimated country-adjusted AUCs for the diagnostic performance of markers in detecting at-risk MASH.

#### Head-to-head comparisons

In an additional head-to-head comparison, we compared the performance of the most frequently used blood-based and imaging biomarkers and scores for detecting MASH and at-risk MASH and fibrosis stages (≥F3 and F4) in a subgroup in which results for all these biomarkers and scores were available, ensuring a sample size of at least 100 to maintain the precision of the analyses. The comparison included FAST, LMS-PDFF, LMS-cT1 and MAST for detecting at-risk MASH and MASH, and VCTE-LSM, MRE, ELF and ADAPT for detecting advanced fibrosis and cirrhosis.

#### Performance of imaging markers at predefined thresholds

We also evaluated the performance of imaging markers using predefined thresholds, reported in the literature, for ruling in at-risk MASH or advanced fibrosis (Supplementary Table 9). Sensitivity and specificity were calculated for each marker at these thresholds within the respective subgroups with available data. Confidence intervals for sensitivity and specificity were calculated using bootstrapping. For each biomarker and threshold, data were resampled with replacement, and sensitivity and specificity were recalculated for each bootstrap iteration.

All statistical analyses were performed using R statistical computing software version 4.3.1.

### Reporting summary

Further information on research design is available in the Nature Portfolio Reporting Summary linked to this article.

## Online content

Any methods, additional references, Nature Portfolio reporting summaries, source data, extended data, supplementary information, acknowledgements, peer review information; details of author contributions and competing interests; and statements of data and code availability are available at 10.1038/s41591-026-04496-2.

## Supplementary information

Supplementary Information Supplementary Tables 1–9, Supplementary Methods and STARD checklist.

Reporting Summary

Peer Review File

## Data Availability

Due to the need to maintain participant confidentiality for this ongoing longitudinal cohort study, and to ensure compliance with varying restrictions across the multiple territories in which participants were recruited, individual participant-level data cannot be placed in the public domain. Qualified researchers may submit requests for deidentified participant data to the corresponding authors, together with a description of the specific data requested, the proposed analyses and the dissemination plan. Requests will be reviewed by the corresponding authors and supported where possible within the necessary legal framework.

## Code availability

All statistical analyses were performed using the R statistical computing software version 4.3.1. The code used for the analyses in this study is available from the corresponding authors upon reasonable request.
