# Supplementary material for: Prospective validation of imaging and serum diagnostic biomarkers of steatohepatitis and fibrosis in MASLD: the LITMUS Imaging Study
Source: Nat Med. 2026 Jul 24;32(8):2959–69. doi: 10.1038/s41591-026-04496-2 (PMC13472969; doi:10.1038/s41591-026-04496-2)
Supplement: Supplementary file 1 [file 41591_2026_4496_MOESM1_ESM.pdf]

nature medicine

Supplementary information

https://doi.org/10.1038/s41591-026-04496-2

Prospective validation of imaging and serum diagnostic biomarkers of steatohepatitis and fibrosis in MASLD: the LITMUS Imaging Study

In the format provided by the authors and unedited

Table of contents

Contents

SUPPLEMENTARY METHODS 2

Magnetic resonance data acquisition 2

Magnetic resonance data analysis 2

SUPPLEMENTARY TABLES 3

Supplementary Table S1. Participant characteristics per country 3

Supplementary Table S2: Head to head comparison of the diagnostic accuracy of PDFF measured using the Liver MultiScan methodology (LMS-PDFF) and PDFF measured using the MRI sequences of each MRI scanner manufacturer (vendor PDFF) in 158 participants where both measures were available. 4

Supplementary Table S3: Accuracy in detecting at-risk MASH, MASH, advanced fibrosis, and cirrhosis with standardization 5

Supplementary Table S4: Accuracy in detecting at-risk MASH, with country adjusted standardization. 6

Supplementary Table S5: Characteristics of the 102 participants included in the head to head comparison for performance of markers for at-risk MASH and the 157 participants included in the head to head comparison for markers of advanced fibrosis 7

Supplementary Table S6: Diagnostic accuracy of selected biomarker threshold for “at risk MASH” in this study, and in the literature 9

Supplementary Table S7: Diagnostic accuracy of selected biomarker thresholds for advanced fibrosis (F3-4) in this study, and the literature 9

Supplementary Table S8: Biomarkers included in the study and descriptions 10

Supplementary Table S9: Markers and predefined thresholds from the literature 12

SUPPLEMENTARY REFERENCES 13

STARD Checklist 16

SUPPLEMENTARY METHODS

Magnetic resonance data acquisition

The procedures for Magnetic resonance data acquisition were standardised in the LITMUS imaging manual that was applied at all sites. Details of the MR acquisition parameters are available in the LITMUS Imaging study protocol[1]. MR data were acquired by MR radiographers who performed liver MRI as part of their clinical practice only once sites fulfilled site technical qualification procedures. MR data were then uploaded to a central secure portal and from there distributed to MR core labs for analysis.

Magnetic resonance data analysis

From the central secure portal, MR data were distributed to four imaging core labs for analysis. Perspectum Ltd (Oxford UK), analysed data for LiverMultiscan PDFF (LMS PDFF) and iron corrected T1 (cT1); Antaros Medical (Mölndal, Sweden) analysed MR elastography (MRE) and Diffusion Weighted Imaging (DWI) data, Resoundant (Rochester, MN, USA) analysed data for PDFF acquired with vendor specific sequences (vPDFF) and the core lab at Seville University in Spain analysed data for DeMILI. Each core lab employed quality control procedures and details of how each biomarker was analysed are available in the study protocol[1], with some more detail for specific biomarkers below.

Liver cT1 was measured using LiverMultiScan, a semi-automated post-processing tool. During image analysis, for all slices acquired, iron-corrected T1 (cT1 maps of the liver were delineated into whole liver segmentation maps using a semi-automatic method.

MRE liver stiffness was measured as the mean shear stiffness within a volume of interest according to the Quantitative Imaging Biomarkers Alliance (QIBA) criteria. Using the MRE magnitude images, the initial VOI was drawn encompassing all visible liver parenchyma up to approximately 1 cm from the liver edge. Non-parenchyma structures and data with potential bias due to visible motion between phase offsets, and that fell outside the 95% confidence interval checkerboard were excluded from the final VOI that was used for to calculate the mean shear stiffness of the liver.

Two methods for measuring proton density fata fraction were employed in our study and some more details on how these were acquired and analysed are provided here. LMS-PDFF, a marker with excellent repeatability and reproducibility, is acquired with a sequence that is standardised across both scanner types (Siemens, GE, Philips) and field strengths (1.5T, 3T), employs AI-driven automated whole-liver segmentation, and core-lab quality control to produce objective, standardized, reproducible PDFF maps independent of vendor-specific differences. Vendor-PDFF is generated from vendor-specific sequences which may or may not require manual analysis and has been shown to have excellent reproducibility across scanner types and field strengths. For this study, vendor-PDFF was analysed in a quality-controlled core lab using Hepatogram+ (Resoundant Inc) to provide objective, standardized, and reproducible measurements from regions of interest covering the right and left lobes of the liver.” A number of exploratory parameters were also captured, including T1 measured by MR manufacturer specific sequences. These fall outside the scope of the current manuscript and will be presented in subsequent publications.

SUPPLEMENTARY TABLES

Supplementary Table S1. Participant characteristics per country

	Finland (N=35)	France (N=76)	Germany (N=3)	Italy (N=16)	Spain (N=88)	Sweden (N=15)	Switzerland (N=4)	UK (N=87)	US (N=33)	Overall (N=357)
**Age (Years)**	50.5 (11.4)	59.2 (11.9)	53.1 (16.4)	51.6 (12.9)	56.6 (12.8)	58.1 (11.9)	54.8 (7.90)	52.9 (15.5)	55.9 (11.3)	55.4 (13.2)
**Sex (Male)** N (%)	11 (31.4%)	43 (56.6%)	1 (33.3%)	7 (43.8%)	53 (60.2%)	9 (60.0%)	4 (100%)	53 (60.9%)	15 (45.5%)	196 (54.9%)
**Ethnicity White** N (%)	35 (100%)	73 (96.1%)	3 (100%)	16 (100%)	87 (98.9%)	15 (100%)	3 (75.0%)	83 (95.4%)	20 (60.6%)	335 (93.8%)
**BMI** Mean (SD**)**	36.7 (4.98)	34.2 (6.22)	35.3 (6.01)	32.1 (2.84)	32.3 (6.00)	31.1 (3.33)	29.5 (4.39)	34.0 (5.19)	36.2 (5.00)	33.8 (5.64)
**Type2 Diabetes** N (%)	10 (28.6%)	45 (59.2%)	2 (66.7%)	3 (18.8%)	49 (55.7%)	11 (73.3%)	0 (0%)	46 (52.9%)	11 (33.3%)	177 (49.6%)
**Hypertension** N (%)	19 (54.3%)	47 (61.8%)	2 (66.7%)	9 (56.3%)	55 (62.5%)	10 (66.7%)	2 (50.0%)	40 (46.0%)	16 (48.5%)	200 (56.0%)
**Advanced Fibrosis** N (%)	4 (11.4%)	47 (61.8%)	2 (66.7%)	5 (31.3%)	45 (51.1%)	7 (46.7%)	2 (50.0%)	39 (44.8%)	15 (45.5%)	166 (46.5%)
**MASH** N (%)	6 (17.1%)	52 (68.4%)	1 (33.3%)	4 (25.0%)	42 (47.7%)	9 (60.0%)	1 (25.0%)	46 (52.9%)	21 (63.6%)	182 (51.0%)
**Significant Fibrosis** N (%)	7 (20.0%)	64 (84.2%)	3 (100%)	9 (56.3%)	66 (75.0%)	12 (80.0%)	3 (75.0%)	73 (83.9%)	19 (57.6%)	256 (71.7%)
**Fibrotic MASH** N (%)	5 (14.3%)	50 (65.8%)	1 (33.3%)	3 (18.8%)	37 (42.0%)	9 (60.0%)	0 (0%)	46 (52.9%)	13 (39.4%)	164 (45.9%)

Supplementary Table S2: Head to head comparison of the diagnostic accuracy of PDFF measured using the Liver MultiScan methodology (LMS-PDFF) and PDFF measured using the MRI sequences of each MRI scanner manufacturer (vendor PDFF) in 158 participants where both measures were available.

Biomarker	At-risk MASH	MASH	Advanced fibrosis	Cirrhosis
LMS-PDFF	0.62 (0.53-0.70)	0.69 (0.61-0.78)	0.52 (0.43-0.61)	0.60 (0.49-0.70)
Vendor-PDFF	0.59 (0.50-0.68)	0.67 (0.58-0.75)	0.55 (0.46-0.64)	0.63 (0.52-0.73)

Abbreviations: AUROC: area under the receiver operating characteristic curve; MASH: metabolic dysfunction associated steatohepatitis, at-risk MASH: MASH and stage F2-4 fibrosis

Supplementary Table S3: Accuracy in detecting at-risk MASH, MASH, advanced fibrosis, and cirrhosis with standardization.

Biomarker		At-risk MASH		MASH		Advanced fibrosis		Cirrhosis	
	n	AUC (95% CI)	Weighted AUC (95% CI)	AUC (95% CI)	Weighted AUC (95% CI)	AUC (95% CI)	Weighted AUC (95% CI)	AUC (95% CI)	Weighted AUC (95% CI)
**MRE**	262	0.74 (0.68-0.80)	0.74 (0.68-0.80)	0.71 (0.65-0.77)	0.71 (0.64-0.77)	0.91 (0.88-0.95)	0.91 (0.88-0.94)	0.91 (0.86-0.95)	0.90 (0.86-0.95)
**LMS-cT1**	252	0.65 (0.59-0.72)	0.66 (0.59-0.72)	0.66 (0.60-0.73)	0.67 (0.60-0.74)	0.59 (0.52-0.66)	0.58 (0.52-0.65)	0.64 (0.55-0.74)	0.65 (0.56-0.74)
**LMS-PDFF**	291	0.65 (0.59-0.72)	0.65 (0.59-0.71)	0.69 (0.63-0.75)	0.69 (0.62-0.75)	0.59 (0.53-0.66)	0.41 (0.34-0.47)	0.67 (0.59-0.75)	0.33 (0.25-0.41)
**deMILI Fibro-MRI**	145	0.50 (0.40-0.59)	0.51 (0.41-0.62)	0.56 (0.47-0.65)	0.58 (0.47-0.68)	0.56 (0.46-0.65)	0.44 (0.33-0.56)	0.50 (0.39-0.62)	0.48 (0.35-0.60)
**deMILI MRI-NASH**	145	0.60 (0.51-0.70)	0.60 (0.50-0.69)	0.63 (0.53-0.72)	0.62 (0.52-0.71)	0.54 (0.44-0.63)	0.53 (0.42-0.63)	0.61 (0.48-0.74)	0.60 (0.45-0.73)
**DWI-ADC**	159	0.60 (0.51-0.69)	0.62 (0.52-0.72)	0.59 (0.50-0.68)	0.60 (0.51-0.70)	0.62 (0.53-0.71)	0.58 (0.49-0.69)	0.61 (0.50-0.73)	0.62 (0.49-0.73)
**VCTE-LSM**	297	0.71 (0.65-0.77)	0.71 (0.65-0.77)	0.68 (0.62-0.74)	0.68 (0.62-0.75)	0.81 (0.76-0.86)	0.81 (0.76-0.85)	0.87 (0.83-0.92)	0.87 (0.82-0.92)
**CAP**	266	0.64 (0.57-0.71)	0.64 (0.57-0.70)	0.66 (0.59-0.72)	0.66 (0.58-0.73)	0.54 (0.47-0.61)	0.54 (0.47-0.62)	0.50 (0.41-0.60)	0.50 (0.41-0.60)
**PRO-C3**	249	0.63 (0.56-0.70)	0.63 (0.56-0.70)	0.63 (0.56-0.70)	0.63 (0.56-0.70)	0.60 (0.52-0.67)	0.59 (0.52-0.66)	0.69 (0.58-0.80)	0.69 (0.56-0.79)
**ALT**	334	0.64 (0.58-0.70)	0.64 (0.58-0.70)	0.65 (0.59-0.71)	0.65 (0.59-0.71)	0.52 (0.45-0.58)	0.48 (0.42-0.55)	0.51 (0.43-0.60)	0.49 (0.41-0.57)
**AST**	334	0.69 (0.63-0.75)	0.69 (0.63-0.75)	0.71 (0.65-0.76)	0.71 (0.65-0.76)	0.58 (0.52-0.64)	0.58 (0.51-0.65)	0.60 (0.52-0.68)	0.60 (0.51-0.67)
**CK18-M30**	238	0.74 (0.67-0.80)	0.73 (0.66-0.80)	0.75 (0.69-0.81)	0.74 (0.68-0.80)	0.63 (0.56-0.70)	0.62 (0.54-0.69)	0.64 (0.54-0.74)	0.64 (0.54-0.75)
**CK18-M65**	235	0.78 (0.72-0.84)	0.76 (0.70-0.83)	0.78 (0.72-0.83)	0.76 (0.70-0.82)	0.66 (0.59-0.73)	0.66 (0.58-0.72)	0.64 (0.55-0.74)	0.64 (0.54-0.74)
**FIB4**	330	0.64 (0.58-0.70)	0.64 (0.58-0.70)	0.63 (0.57-0.69)	0.63 (0.57-0.69)	0.74 (0.69-0.79)	0.74 (0.69-0.79)	0.78 (0.72-0.84)	0.78 (0.71-0.83)
**ADAPT**	246	0.65 (0.58-0.72)	0.65 (0.58-0.73)	0.64 (0.57-0.71)	0.64 (0.57-0.71)	0.76 (0.71-0.82)	0.77 (0.70-0.82)	0.80 (0.72-0.89)	0.80 (0.71-0.88)
**FAST**	189	0.73 (0.66-0.80)	0.73 (0.65-0.80)	0.73 (0.66-0.80)	0.73 (0.66-0.80)	0.69 (0.61-0.76)	0.69 (0.61-0.76)	0.76 (0.67-0.85)	0.75 (0.66-0.85)
**ELF**	275	0.67 (0.60-0.73)	0.66 (0.60-0.72)	0.65 (0.59-0.72)	0.65 (0.58-0.71)	0.74 (0.68-0.80)	0.74 (0.68-0.79)	0.82 (0.75-0.89)	0.82 (0.74-0.88)
**NIS2+**	220	0.82 (0.77-0.88)	0.81 (0.75-0.87)	0.83 (0.77-0.88)	0.82 (0.75-0.87)	0.70 (0.63-0.77)	0.69 (0.62-0.76)	0.63 (0.53-0.73)	0.61 (0.51-0.72)
**Agile 3+**	288	0.68 (0.62-0.74)	0.68 (0.61-0.73)	0.65 (0.58-0.71)	0.64 (0.58-0.71)	0.84 (0.80-0.89)	0.84 (0.80-0.88)	0.89 (0.84-0.94)	0.89 (0.84-0.94)
**Agile 4**	288	0.66 (0.60-0.72)	0.66 (0.60-0.72)	0.63 (0.57-0.70)	0.63 (0.57-0.69)	0.82 (0.77-0.87)	0.82 (0.77-0.87)	0.88 (0.83-0.94)	0.89 (0.83-0.93)
**cTAG**	186	0.70 (0.63-0.78)	0.72 (0.64-0.79)	0.71 (0.64-0.79)	0.73 (0.65-0.80)	0.69 (0.61-0.76)	0.69 (0.61-0.76)	0.71 (0.59-0.82)	0.70 (0.59-0.81)
**MEFIB**	251	0.64 (0.59-0.70)	0.64 (0.59-0.69)	0.62 (0.57-0.68)	0.62 (0.57-0.68)	0.72 (0.67-0.77)	0.72 (0.67-0.77)	0.76 (0.69-0.84)	0.76 (0.68-0.84)
**MAST**	202	0.78 (0.71-0.84)	0.78 (0.71-0.84)	0.77 (0.71-0.84)	0.78 (0.71-0.85)	0.78 (0.72-0.84)	0.78 (0.71-0.84)	0.80 (0.72-0.87)	0.81 (0.72-0.88)

Weights based on the inverse of the propensity based on age, sex, BMI, diabetes, and fibrosis stage. Sample sizes correspond to subsets with marker results and propensity data.

Supplementary Table S4: Accuracy in detecting at-risk MASH, with country adjusted standardization.

Biomarker	At-risk MASH	
	AUROC (95% CI)	Country adjusted AUROC (95% CI)
MRE	0.75 (0.69-0.81)	0.69 (0.61-0.75)
LMS-cT1	0.66 (0.59-0.72)	0.68 (0.61-0.75)
LMS-PDFF	0.66 (0.59-0.72)	0.65 (0.58-0.71)
Vendor-PDFF	0.59 (0.51-0.68)	0.58 (0.49-0.66)
deMILI Fibro-MRI	0.49 (0.40-0.58)	0.57 (0.47-0.66)
deMILI MRI-NASH	0.62 (0.53-0.71)	0.63 (0.54-0.72)
DWI-ADC	0.60 (0.51-0.69)	0.40 (0.31-0.51)
VCTE-LSM	0.71 (0.65-0.77)	0.67 (0.60-0.73)
CAP	0.64 (0.58-0.71)	0.64 (0.57-0.72)
PRO-C3	0.63 (0.56-0.70)	0.60 (0.53-0.67)
ALT	0.64 (0.58-0.70)	0.63 (0.56-0.69)
AST	0.69 (0.63-0.75)	0.69 (0.63-0.75)
CK18-M30	0.74 (0.68-0.80)	0.72 (0.65-0.79)
CK18-M65	0.78 (0.72-0.84)	0.75 (0.68-0.81)
FIB4	0.64 (0.58-0.70)	0.64 (0.57-0.70)
ADAPT	0.65 (0.58-0.72)	0.61 (0.54-0.69)
FAST	0.73 (0.66-0.80)	0.68 (0.59-0.77)
ELF	0.67 (0.60-0.73)	0.65 (0.58-0.72)
NIS2+	0.82 (0.77-0.88)	0.82 (0.75-0.87)
Agile 3+	0.68 (0.62-0.74)	0.63 (0.56-0.69)
Agile 4	0.66 (0.60-0.72)	0.65 (0.58-0.71)
cTAG	0.70 (0.63-0.78)	0.69 (0.61-0.77)
MEFIB	0.64 (0.59-0.70)	0.53 (0.46-0.61)
MAST	0.78 (0.71-0.84)	0.77 (0.69-0.83)

Supplementary Table S5: Characteristics of the 102 participants included in the head to head comparison for performance of markers for at-risk MASH and the 157 participants included in the head to head comparison for markers of advanced fibrosis

	**Subgroup for MASH Markers (N=102)**	**Subgroup for Fibrosis Markers (N=157)**
**Age (Years),** Mean (SD)	57.59 (13.93)	55.42 (13.49)
**Sex (Male)**	50 (49.0%)	84 (53.5%)
**Ethnicity (**White	100 (98.0%)	154 (98.1%)
**BMI** (kg/m^2)	32.59 (5.24)	33.89 (5.13)
**ALT (U/L)**	53.34 (41.90)	58.46 (52.76)
**AST (U/L)**	44.37 (29.13)	45.40 (29.96)
**GGT (U/L)**	103.93 (155.93)	87.04 (111.81)
**Platelet (10^9/L)**	225.63 (63.37)	226.66 (69.52)
**Albumin (g/dL)**	4.39 (0.44)	4.23 (0.43)
**Glucose (mmol/L)**	6.50 (2.68)	6.57 (2.34)
**Total cholesterol (mmol/L)**	4.91 (1.34)	4.70 (1.32)
**Triglyceride (mmol/L)**	2.00 (0.95)	1.95 (0.91)
**Diabetes**	53 (52.0%)	75 (47.8%)
**Hypertension**	58 (56.9%)	94 (59.9%)
**Histology**		
**MASH**	56 (54.9%)	78 (49.7%)
**Steatosis**		
0	10 (9.8%)	20 (12.7%)
1	39 (38.2%)	53 (33.8%)
2	33 (32.4%)	49 (31.2%)
3	20 (19.6%)	35 (22.3%)
**Inflammation**		

	Subgroup for MASH Markers (N=102)	Subgroup for Fibrosis Markers (N=157)
0	12 (11.8%)	28 (17.8%)
1	72 (70.6%)	97 (61.8%)
2	17 (16.7%)	27 (17.2%)
3	1 (1.0%)	5 (3.2%)
**Ballooning**		
0	34 (33.3%)	63 (40.1%)
1	34 (33.3%)	58 (36.9%)
2	34 (33.3%)	36 (22.9%)
**Fibrosis Score**		
0	6 (5.9%)	25 (15.9%)
1	12 (11.8%)	21 (13.4%)
2	30 (29.4%)	42 (26.8%)
3	36 (35.3%)	48 (30.6%)
4	18 (17.6%)	21 (13.4%)
**NAS Score**		
0	5 (4.9%)	13 (8.3%)
1	11 (10.8%)	19 (12.1%)
2	7 (6.9%)	12 (7.6%)
3	19 (18.6%)	31 (19.7%)
4	23 (22.5%)	25 (15.9%)
5	21 (20.6%)	32 (20.4%)
6	15 (14.7%)	20 (12.7%)
7	1 (1.0%)	4 (2.5%)
8	0 (0.0%)	1 (0.6%)

Supplementary Table S6: Diagnostic accuracy of selected biomarker threshold for "at risk MASH" in this study, and in the literature

Biomarker	Threshold	Study	n	Sensitivity	Specificity
MRE (kPa)	3.3	LITMUS	275	0.69	0.76
		Li et al[2]	89	0.79	0.82
LMS-cT1 (ms)	875	LITMUS	265	0.58	0.61
		Andersson et al[3]	543	0.59	0.81
NIS2+	0.68	LITMUS	221	0.72	0.81
		Harrison et al[4]	2035	0.62	0.85
FAST	0.67	LITMUS	190	0.41	0.86
		Newsome et al[5]	1026	0.49	0.92
cTAG	0.34	LITMUS	187	0.55	0.76
		Dennis et al[6]	86	0.92	0.79
MAST	0.242	LITMUS	203	0.41	0.91
		Noureddin et al[7]	42	0.75	0.90

Supplementary Table S7: Diagnostic accuracy of selected biomarker thresholds for advanced fibrosis (F3-4) in this study, and the literature

Biomarker	Threshold	study	n	Sensitivity	Specificity
MRE (kPa)	3.5	LITMUS	275	0.71	0.92
		Liang et al[8]	798	0.87	0.88
VCTE-LSM (kPa)	10	LITMUS	298	0.73	0.74
		Mozes et al[9]	5489	0.71	0.82
Agile 3+	0.679	LITMUS	288	0.65	0.84
		Dalbeni et al[10]	6955	0.68	0.87
Agile 4	0.545	LITMUS	288	0.20	0.99
		Malandris et al[11]	6037	0.67	0.93
MEFIB	MRE≥3.3 and FIB4≥1.6*	LITMUS	252	0.51	0.93
		Jung J et al[12]	238	PPV 0.97 and 0.91	NPV 0.83 and 0.59

Supplementary Table S8: Biomarkers included in the study and descriptions

	Biomarker	Details	Ref
Magnetic Resonance markers	**MRE**	Liver stiffness measurement (LSM) obtained with magnetic resonance elastography, (Resoundant, USA, licensed to major MRI manufacturers.)	13
	**LMS-cT1**	Liver MultiScan corrected T1 (corrected for iron concentration and standardized to 3T field strength).	14, 15
	**MR-PDFF / LMS-PDFF**	Proton density fat fraction represents the percentage of fat signal in the imaged tissue, correlating with hepatic steatosis.	16, 17
	**deMILI Fibrosis-MRI**	Fibrosis component of DeMILI, predicting fibrosis from radiomics features	18
	**deMILI NASH-MRI**	NASH component of DeMILI, predicting NASH from radiomics features.	18
	**DWI_ADC**	Diffusion-weighted imaging apparent diffusion coefficient is a measure of how freely water molecules can travel in tissue microenvironments.	19
Ultrasound elastography markers	**VCTE-LSM**	Liver stiffness measurement (LSM) by vibration-controlled transient elastography (VCTE) measured by FibroScan (Echosens)	20, 21
	**CAP**	Controlled Attenuation Parameter measured by FibroScan (Echosens)	21
Single blood-based markers	**PROC3**	A neo-epitope marker detecting the procollagen type III N-terminal fragment of P3NP has been identified as a by-product of the cleavage of procollagen III to produce collagen III. It has been known since then as a marker of liver fibrosis and has been validated among many other markers to be the one most constantly associated with steatosis as well as non-alcoholic steatohepatitis (MASH)	22, 23
	**ALT**	Alanine transaminase	
	**AST**	Aspartate aminotransferase	
	**CK-18 M30/ M65**	Cytokeratins are keratin-containing proteins that form intermediate filaments and comprise the structure of cytoskeletons of epithelial cells. CK18 is found predominantly in glandular epithelia of the digestive, respiratory, and urogenital tracts. It is the major intermediate filament protein of the liver. During apoptosis of hepatocytes, caspases	24, 25

		cleave CK18 generating fragments that can be detectable using immunoassays. It is one of the most widely investigated biomarkers for MASH as a stand-alone test or as part of prediction models. The two main enzyme assays of CK18 that have been studied are M30 and M65, which supposedly measure hepatocyte apoptosis and total cell death, respectively.	
Multi-markers scores that include only blood-based marker data	**FIB4**	age x ast / (platelet x sqrt(alt))	26
	**ADAPT**	ADAPT = exp(log10((Age x Pro-C3)/sqrt(Platelets))) + Diabetes	27
	**ELF**	Using the ADVIA Centaur and ADVIA Centaur XP/XPT Systems as an advanced automated immunoassay analyzer: 2.278 + 0.851 ln(HA) + 0.751 ln(PIIINP) + 0.394 ln(TIMP1)	28
	**NIS2+**	NIS2+ is an optimization of NIS4, specifically designed to improve score robustness for at-risk MASH. It is based on two biomarkers: miR-34a-5p and YKL-40	29, 30
Composite scores – include data from imaging and blood-based markers	**FAST**	Liver stiffness measurement (LSM) by vibration-controlled transient elastography (VCTE) and ultrasound attenuation (Controlled Attenuation Parameters, CAP) both measured by FibroScan (Echosens) combined with AST<br>1 / (1 + exp(-A)) A=(-1.65 +1.07 x ln(LSM) + 2.66×10^{-8} x CAP^3 -63.3 x AST^{-1})	5
	**Agile 3+**	Liver stiffness measurement (LSM) by vibration-controlled transient elastography (VCTE) measured by FibroScan (Echosens) combined with AST, ALT, platelet count (PLT), presence of diabetes (DIAB), sex and age.<br>1 / (1 + exp(-A)) A=(-3.92368+2.29714 x ln(LSM)-0.00902 x PLT-0.98633 x (ALT/AST)+1.08636 x DIAB -0.38581 x sex +0.03018 x age)	31
	**Agile 4**	Liver stiffness measurement (LSM) by vibration-controlled transient elastography (VCTE) measured by FibroScan (Echosens) combined with AST, ALT, platelet count (PLT), presence of diabetes (DIAB) and sex.<br>1 / (1 + exp(-A)) A=(7.50139-15.42498 x 1/√(LSM) - 0.01378 x PLT -1.41149 x (ALT/AST) - 0.53281 x sex + 0.41741 x DIAB)	31

	**cTAG**	cTAG = exp(-14.3 + 0.00807*cT1 + 0.639*Gluc + 0.0519*ast)/1+ exp(-14.3 + 0.00807*cT1 + 0.639*Gluc + 0.0519*ast)	32
	**MEFIB**	MEFIB is an index that is defined as FIB-4 ≥1.6 and MRE ≥3.3kPa	12
	**MAST**	Computed from MRE-based liver stiffness measurement (LSM), MR-PDFF and serum AST. MAST = -12.17 + 7.07 log MRE + 0.037 PDFF + 3.55 log AST	7

Supplementary Table S9: Markers and predefined thresholds from the literature

At-risk MASH markers:	Evaluated threshold	Fibrosis markers:	Evaluated threshold (advanced fibrosis)
MRE	3.3[2]	MRE	3.5kPa[8]
LMS-CT1	875ms[3]	deMILI Fibro MRI	0.5[33]
LMS-PDFF	8%[34]	VCTE-LSM	10kPa[9, 35]
deMILI NASH-MRI	0.5[33]	Agile 3+	0.679
CAP	280 dB/m[34]	Agile 4	0.565
NIS2+	0.68[4]	MEFIB	MRE≥3.3 and FIB4≥1.6[12]
FAST	0.67[5]		
cTAG (%)	34%[6]		
MAST	0.242[36]		

These thresholds relate to the diagnostic context of use and do not address test reproducibility, which was outside the scope of this work and has been addressed elsewhere[37-39].

SUPPLEMENTARY REFERENCES

1. Pavlides M, Mózes FE, Akhtar S, et al. Liver investigation: Testing marker utility in steatohepatitis (LITMUS): Assessment & validation of imaging modality performance across the NAFLD spectrum in a prospectively recruited cohort study (the LITMUS imaging study): Study protocol. Contemporary Clinical Trials 2023;134:107352.
2. Li J, Lu X, Zhu Z, et al. Head-to-head comparison of magnetic resonance elastography-based liver stiffness, fat fraction, and T1 relaxation time in identifying at-risk NASH. Hepatology 2023;78:1200-1208.
3. Andersson A, Kelly M, Imajo K, et al. Clinical Utility of MRI Biomarkers for Identifying NASH Patients at High Risk of Progression: A Multicenter Pooled Data and Meta-Analysis. Clin Gastroenterol Hepatol 2021.
4. Harrison SA, Ratziu V, Magnanensi J, et al. NIS2+™, an optimisation of the blood-based biomarker NIS4® technology for the detection of at-risk NASH: A prospective derivation and validation study. J Hepatol 2023;79:758-767.
5. Newsome PN, Sasso M, Deeks JJ, et al. FibroScan-AST (FAST) score for the non-invasive identification of patients with non-alcoholic steatohepatitis with significant activity and fibrosis: a prospective derivation and global validation study. Lancet Gastroenterol Hepatol 2020;5:362-373.
6. Dennis A, Mouchti S, Kelly M, et al. A composite biomarker using multiparametric magnetic resonance imaging and blood analytes accurately identifies patients with non-alcoholic steatohepatitis and significant fibrosis. Scientific reports 2020;10:1-11.
7. Noureddin M, Truong E, Gornbein JA, et al. MRI-based (MAST) score accurately identifies patients with NASH and significant fibrosis. J Hepatol 2022;76:781-787.
8. Liang J-x, Ampuero J, Niu H, et al. An individual patient data meta-analysis to determine cut-offs for and confounders of NAFLD-fibrosis staging with magnetic resonance elastography. Journal of Hepatology 2023;79:592-604.
9. Mózes FE, Lee JA, Selvaraj EA, et al. Diagnostic accuracy of non-invasive tests for advanced fibrosis in patients with NAFLD: an individual patient data meta-analysis. Gut 2021.
10. Dalbeni A, Lombardi R, Henrique M, et al. Diagnostic accuracy of AGILE 3+ score for advanced fibrosis in patients with NAFLD: A systematic review and meta-analysis. Hepatology 2024;79:1107-1116.
11. Malandris K, Katsoula A, Liakos A, et al. Diagnostic accuracy of Agile-4 score for liver cirrhosis in patients with metabolic dysfunction-associated steatotic liver disease. A systematic review and meta-analysis of diagnostic test accuracy studies. Diabetes Obes Metab 2025;27:1406-1414.
12. Jung J, Loomba RR, Imajo K, et al. MRE combined with FIB-4 (MEFIB) index in detection of candidates for pharmacological treatment of NASH-related fibrosis. Gut 2021;70:1946-1953.
13. Loomba R, Wolfson T, Ang B, et al. Magnetic resonance elastography predicts advanced fibrosis in patients with nonalcoholic fatty liver disease: a prospective study. Hepatology 2014;60:1920-8.
14. Banerjee R, Pavlides M, Tunnicliffe EM, et al. Multiparametric magnetic resonance for the non-invasive diagnosis of liver disease. J Hepatol 2014;60:69-77.
15. Pavlides M, Banerjee R, Tunnicliffe EM, et al. Multiparametric magnetic resonance imaging for the assessment of non-alcoholic fatty liver disease severity. Liver Int 2017;37:1065-1073.
16. Caussy C, Alquiraish MH, Nguyen P, et al. Optimal threshold of controlled attenuation parameter with MRI-PDFF as the gold standard for the detection of hepatic steatosis. Hepatology 2018;67:1348-1359.
17. Dennis A, Kelly MD, Fernandes C, et al. Correlations Between MRI Biomarkers PDFF and cT1 With Histopathological Features of Non-Alcoholic Steatohepatitis. Front Endocrinol (Lausanne) 2020;11:575843.

18. Gallego-Duran R, Cerro-Salido P, Gomez-Gonzalez E, et al. Imaging biomarkers for steatohepatitis and fibrosis detection in non-alcoholic fatty liver disease. Sci Rep 2016;6:31421.
19. Murphy P, Hooker J, Ang B, et al. Associations between histologic features of nonalcoholic fatty liver disease (NAFLD) and quantitative diffusion-weighted MRI measurements in adults. J Magn Reson Imaging 2015;41:1629-38.
20. Siddiqui MS, Vuppalanchi R, Van Natta ML, et al. Vibration-Controlled Transient Elastography to Assess Fibrosis and Steatosis in Patients With Nonalcoholic Fatty Liver Disease. Clin Gastroenterol Hepatol 2019;17:156-163 e2.
21. Eddowes PJ, Sasso M, Allison M, et al. Accuracy of FibroScan Controlled Attenuation Parameter and Liver Stiffness Measurement in Assessing Steatosis and Fibrosis in Patients With Nonalcoholic Fatty Liver Disease. Gastroenterology 2019;156:1717-1730.
22. Nielsen MJ, Nedergaard AF, Sun S, et al. The neo-epitope specific PRO-C3 ELISA measures true formation of type III collagen associated with liver and muscle parameters. Am J Transl Res 2013;5:303-15.
23. Boyle M, Tiniakos D, Schattenberg JM, et al. Performance of the PRO-C3 collagen neo-epitope biomarker in non-alcoholic fatty liver disease. JHEP Rep 2019;1:188-198.
24. Feldstein AE, Wieckowska A, Lopez AR, et al. Cytokeratin-18 fragment levels as noninvasive biomarkers for nonalcoholic steatohepatitis: a multicenter validation study. Hepatology 2009;50:1072-8.
25. Lee J, Vali Y, Boursier J, et al. Accuracy of cytokeratin 18 (M30 and M65) in detecting non-alcoholic steatohepatitis and fibrosis: A systematic review and meta-analysis. PLoS One 2020;15:e0238717.
26. Sterling RK, Lissen E, Clumeck N, et al. Development of a simple noninvasive index to predict significant fibrosis in patients with HIV/HCV coinfection. Hepatology 2006;43:1317-25.
27. Daniels SJ, Leeming DJ, Eslam M, et al. ADAPT: An Algorithm Incorporating PRO-C3 Accurately Identifies Patients With NAFLD and Advanced Fibrosis. Hepatology 2019;69:1075-1086.
28. Vali Y, Lee J, Boursier J, et al. Enhanced liver fibrosis test for the non-invasive diagnosis of fibrosis in patients with NAFLD: A systematic review and meta-analysis. J Hepatol 2020;73:252-262.
29. Harrison SA, Ratziu V, Magnanensi J, et al. NIS2+, an optimisation of the blood-based biomarker NIS4(R) technology for the detection of at-risk NASH: A prospective derivation and validation study. J Hepatol 2023;79:758-767.
30. Ratziu V, Harrison SA, Hajji Y, et al. NIS2+TM as a screening tool to optimize patient selection in metabolic dysfunction-associated steatohepatitis clinical trials. Journal of Hepatology 2024;80:209-219.
31. Sanyal AJ, Foucquier J, Younossi ZM, et al. Enhanced diagnosis of advanced fibrosis and cirrhosis in individuals with NAFLD using FibroScan-based Agile scores. J Hepatol 2023;78:247-259.
32. Dennis A, Mouchti S, Kelly M, et al. A composite biomarker using multiparametric magnetic resonance imaging and blood analytes accurately identifies patients with non-alcoholic steatohepatitis and significant fibrosis. Sci Rep 2020;10:15308.
33. Gallego-Duran R, Ranchal I, Rico MC, et al. Non-invasive management of non-alcoholic fatty liver disease. Journal of Hepatology 2014;60.
34. Harrison SA, Bedossa P, Guy CD, et al. A Phase 3, Randomized, Controlled Trial of Resmetirom in NASH with Liver Fibrosis. N Engl J Med 2024;390:497-509.
35. Mózes FE, Lee JA, Vali Y, et al. Performance of non-invasive tests and histology for the prediction of clinical outcomes in patients with non-alcoholic fatty liver disease: an individual participant data meta-analysis. Lancet Gastroenterol Hepatol 2023;8:704-713.

36. Noureddin M, Truong E, Gornbein JA, et al. MRI-based (MAST) score accurately identifies patients with NASH and significant fibrosis. J Hepatol 2021.
37. Ndaa M, Pandya PK, Swensson J, et al. Reliable Monitoring of Patients With Metabolic Dysfunction-Associated Steatotic Liver Disease Using Imaging: A Systematic Literature Review and Meta-Analysis on Measurement Repeatability. Endocrine Practice.
38. Pierce TT, Ozturk A, Sherlock SP, et al. Reproducibility and Repeatability of US Shear-Wave and Transient Elastography in Nonalcoholic Fatty Liver Disease. Radiology 2024;312:e233094.
39. Siddiqi H, Huang DQ, Mittal N, et al. Repeatability of vibration-controlled transient elastography versus magnetic resonance elastography in patients with cirrhosis: A prospective study. Aliment Pharmacol Ther 2024;60:484-491.

STARD Checklist

Section & Topic	No	Item	Reported on page #
**TITLE OR ABSTRACT**			
	**1**	Identification as a study of diagnostic accuracy using at least one measure of accuracy<br>(such as sensitivity, specificity, predictive values, or AUC)	1, 4
**ABSTRACT**			
	**2**	Structured summary of study design, methods, results, and conclusions<br>(for specific guidance, see STARD for Abstracts)	4
**INTRODUCTION**			
	**3**	Scientific and clinical background, including the intended use and clinical role of the index test	5-6
	**4**	Study objectives and hypotheses	5-6
**METHODS**			
Study design	**5**	Whether data collection was planned before the index test and reference standard<br>were performed (prospective study) or after (retrospective study)	12
Participants	**6**	Eligibility criteria	12
	**7**	On what basis potentially eligible participants were identified<br>(such as symptoms, results from previous tests, inclusion in registry)	12
	**8**	Where and when potentially eligible participants were identified (setting, location and dates)	12
	**9**	Whether participants formed a consecutive, random or convenience series	12
Test methods	**10a**	Index test, in sufficient detail to allow replication	13-14
	**10b**	Reference standard, in sufficient detail to allow replication	12
	**11**	Rationale for choosing the reference standard (if alternatives exist)	N/A no alternative reference standard exists
	**12a**	Definition of and rationale for test positivity cut-offs or result categories<br>of the index test, distinguishing pre-specified from exploratory	16-17
	**12b**	Definition of and rationale for test positivity cut-offs or result categories<br>of the reference standard, distinguishing pre-specified from exploratory	15
	**13a**	Whether clinical information and reference standard results were available<br>to the performers/readers of the index test	13
	**13b**	Whether clinical information and index test results were available<br>to the assessors of the reference standard	12
Analysis	**14**	Methods for estimating or comparing measures of diagnostic accuracy	15-16
	**15**	How indeterminate index test or reference standard results were handled	n/a
	**16**	How missing data on the index test and reference standard were handled	n/a
	**17**	Any analyses of variability in diagnostic accuracy, distinguishing pre-specified from exploratory	n/a
	**18**	Intended sample size and how it was determined	n/a
**RESULTS**			
Participants	**19**	Flow of participants, using a diagram	Extended data Figure 1
	**20**	Baseline demographic and clinical characteristics of participants	Table 1
	**21a**	Distribution of severity of disease in those with the target condition	Table 1
	**21b**	Distribution of alternative diagnoses in those without the target condition	N/A
	**22**	Time interval and any clinical interventions between index test and reference standard	7

Test results	**23**	Cross tabulation of the index test results (or their distribution) by the results of the reference standard	Table 2
	**24**	Estimates of diagnostic accuracy and their precision (such as 95% confidence intervals)	Figures 1 and 2
	**25**	Any adverse events from performing the index test or the reference standard	N/A
**DISCUSSION**			
	**26**	Study limitations, including sources of potential bias, statistical uncertainty, and generalisability	10-15
	**27**	Implications for practice, including the intended use and clinical role of the index test	10-15
**OTHER INFORMATION**			
	**28**	Registration number and name of registry	30
	**29**	Where the full study protocol can be accessed	Cited on page 30
	**30**	Sources of funding and other support; role of funders	16
