# Supplementary material for: Prospective validation of imaging and serum diagnostic biomarkers of steatohepatitis and fibrosis in MASLD: the LITMUS Imaging Study
Source: Nat Med. 2026 Jul 24;32(8):2959–69. doi: 10.1038/s41591-026-04496-2 (PMC13472969; doi:10.1038/s41591-026-04496-2)
Supplement: Supplementary file 2 [file 41591_2026_4496_MOESM2_ESM.pdf]

nature portfolio

Corresponding author(s): Michael Pavlides, Quentin Anstee

Last updated by author(s): May 12, 2026

Reporting Summary

Nature Portfolio wishes to improve the reproducibility of the work that we publish. This form provides structure for consistency and transparency in reporting. For further information on Nature Portfolio policies, see our Editorial Policies and the Editorial Policy Checklist.

Statistics

For all statistical analyses, confirm that the following items are present in the figure legend, table legend, main text, or Methods section.

n/a	Confirmed	
☐	☒	The exact sample size (*n*) for each experimental group/condition, given as a discrete number and unit of measurement
☐	☒	A statement on whether measurements were taken from distinct samples or whether the same sample was measured repeatedly
☐	☒	The statistical test(s) used AND whether they are one- or two-sided<br>*Only common tests should be described solely by name; describe more complex techniques in the Methods section.*
☐	☒	A description of all covariates tested
☐	☒	A description of any assumptions or corrections, such as tests of normality and adjustment for multiple comparisons
☐	☒	A full description of the statistical parameters including central tendency (e.g. means) or other basic estimates (e.g. regression coefficient) AND variation (e.g. standard deviation) or associated estimates of uncertainty (e.g. confidence intervals)
☐	☒	For null hypothesis testing, the test statistic (e.g. *F*, *t*, *r*) with confidence intervals, effect sizes, degrees of freedom and *P* value noted<br>*Give P values as exact values whenever suitable.*
☒	☐	For Bayesian analysis, information on the choice of priors and Markov chain Monte Carlo settings
☒	☐	For hierarchical and complex designs, identification of the appropriate level for tests and full reporting of outcomes
☐	☒	Estimates of effect sizes (e.g. Cohen's *d*, Pearson's *r*), indicating how they were calculated

Our web collection on statistics for biologists contains articles on many of the points above.

Software and code

Policy information about availability of computer code

Data collection	A purpose-built MySQL relational database was used for data capture. The web-accessible secure front-end database comprises 14 related electronic clinical record form (eCRF) data-tables and contains around 1200 data fields, equating to approximately 1–2 megabytes of data per subject and is designed to fulfill the regulatory requirements for record keeping. The database is configured in a Clinical Data Interchange Standards Consortium (CDISC, www.cdisc.org) compliant format to aid information system interoperability for future collaborative projects.
Data analysis	All statistical analyses were performed using R statistical computing software version 4.3.1 (Vienna, Austria).

For manuscripts utilizing custom algorithms or software that are central to the research but not yet described in published literature, software must be made available to editors and reviewers. We strongly encourage code deposition in a community repository (e.g. GitHub). See the Nature Portfolio guidelines for submitting code & software for further information.

Data

Policy information about availability of data

All manuscripts must include a data availability statement. This statement should provide the following information, where applicable:

- Accession codes, unique identifiers, or web links for publicly available datasets
- A description of any restrictions on data availability
- For clinical datasets or third party data, please ensure that the statement adheres to our policy

Due to the need to maintain participant confidentiality for this ongoing longitudinal cohort study, and to ensure compliance with varying restrictions across the

multiple territories in which participants were recruited, individual participant level data can not be placed in the public domain. Qualified researchers may submit requests for deidentified participant data to the corresponding authors, together with a description of the specific data requested, the proposed analyses and the dissemination plan. Requests will be reviewed by the corresponding authors and supported where possible within the necessary legal framework.

Research involving human participants, their data, or biological material

Policy information about studies with human participants or human data. See also policy information about sex, gender (identity/presentation), and sexual orientation and race, ethnicity and racism.

Reporting on sex and gender	Data was only collected for the self reported sex of participants.<br>Sex was considered in the study and participants were recruited irrespective of sex and analysis adjusting for variables including sex was conducted.
Reporting on race, ethnicity, or other socially relevant groupings	Data was collected on the self reported ethnicity of participants.
Population characteristics	A total of 357 participants with a mean age of 55.4 years were included; 196 were of male sex (54.9%), 335 (98%) were of white ethnicity, mean BMI was 33.8kg/m2, 177 (49.6%) had diabetes, 200 (56%) had hypertension.
Recruitment	Participants were recruited sequentially.<br>All participants gave written informed consent for participation in the study.<br>The participants were reimbursed for travel expenses and food for attending research imaging assessments. These assessments were not part of routine clinical care and participants were asked to come for their scan having fasted for at least 4 hours.
Ethics oversight	The study was conducted in line with the ethical principles for medical research involving human subjects, as specified in the Declaration of Helsinki. The project was approved by the relevant Ethical Committees in the participating countries and all participants provided written informed consent prior to inclusion. In the UK, the LITMUS Imaging Study was approved by the London Queen Square Research Ethics Committee (18/LO/1953), in France by Comite de protection des personnes, Sud Mediterranee IV (Reference CPP: 19 06 04; ID-RCB:2019-A01308-49) for Paris, and Comite de Protection des Personnes (CPP) Ouest II ,Angers (CPP #: CB-2010-01, Dossier #: DC-2011-1467, Dossier #: AC 2014-2329) for Angers, in Spain by CEI de los Hospitales Universitarios Virgen Macarena y Virgen del Rocío (Informe Dictamen Favorable, Proyecto Investigación Biomédica, C.P. LITMUS_GA_777377 - C.I, 20 de Septiembre de 2018) for Seville, Drug Research Ethics (Vall d'Hebron) for Barcelona (PR(AG)507/2020) and CEIm Area de Salud Valladolid Este, University Hospital Valladolid (18-1173) for Valladolid, in the USA initial approval was given by Integ Review IRB (Protocol Number: LI-001-2019) and continued approval by ADVARRA IRB (CONTINUING REVIEW APPROVAL CR00408748), in Italy by A.O.U. City of Health and Science of Turin - A.O. Mauritian Order - ASL TO 1 (Prot N. N 0125391 18 Dec 2018) for Torino, and Comitato Etico Palermo 1, Azienda Ospedaiera Universitaria Policliniclo Paolo Giaccone di Palermo (Verbale N. 11/2019, 16.12.2019<br>) for Palermo, in Germany by Ethikkommission der Landesärztekammer Rheinland-Pfalz (2018-13269_1, 19.08/2018), in Sweden by Regionala etikpövningsnämnden I Linköping (Dnr 2018/393-31<br>), in Switzerland by Kantonale Ethikkommission (KEK), Bern (KEK Nr 2019-00609) and in Finland by HUS eettinen toimikunta IV (HUS/1784/2019).

Note that full information on the approval of the study protocol must also be provided in the manuscript.

Field-specific reporting

Please select the one below that is the best fit for your research. If you are not sure, read the appropriate sections before making your selection.

☒ Life sciences ☐ Behavioural & social sciences ☐ Ecological, evolutionary & environmental sciences

For a reference copy of the document with all sections, see nature.com/documents/nr-reporting-summary-flat.pdf

Life sciences study design

All studies must disclose on these points even when the disclosure is negative.

Sample size	A formal sample size calculation was not undertaken prior to the study. The maximum number of participants were recruited from this nested biomarker study and this study represents the largest of its kind.
Data exclusions	data were excluded for cases without paired biopsy and biomarker data within 6 months of each other. Exclusion of data according to these criteria was pre-defined and it was done in order to minimise potential bias related to the time lapse between the reference standard (biopsy) and biomarker acquisition
Replication	All MRI biomarkers were reported centrally by qualified imaging core labs. All serum based biomarkers were measured in central qualified laboratories. To ensure reproducibility in the reporting of the reference standard (liver biopsy histological scoring), individual biopsies were scored by consensus between two expert pathologists. and was examined by previous studies and in a separate study by our group.Reproducibility of biomarkers was not examined for the purposes of this paper.

Randomization	no randomisation was applied as this was a single arm study
Blinding	all analyses of biomarkers wereconducted by investigators blinded to the reference standard. Analysis of the reference standard was conducted by investigators blinded to the biomarkers. All investigators were blinded to demographic and other clinical data.

Reporting for specific materials, systems and methods

We require information from authors about some types of materials, experimental systems and methods used in many studies. Here, indicate whether each material, system or method listed is relevant to your study. If you are not sure if a list item applies to your research, read the appropriate section before selecting a response.

Materials & experimental systems

n/a	Involved in the study
☒	☐ Antibodies
☒	☐ Eukaryotic cell lines
☒	☐ Palaeontology and archaeology
☒	☐ Animals and other organisms
☐	☒ Clinical data
☒	☐ Dual use research of concern
☒	☐ Plants

Methods

n/a	Involved in the study
☒	☐ ChIP-seq
☒	☐ Flow cytometry
☒	☐ MRI-based neuroimaging

Clinical data

Policy information about clinical studies

All manuscripts should comply with the ICMJE guidelines for publication of clinical research and a completed CONSORT checklist must be included with all submissions.

Clinical trial registration	NCT05479721
Study protocol	https://doi.org/10.1016/j.cct.2023.107352
Data collection	Data were collected in secondary and tertiary care centers in Europe and the USA between 2018 and 2023.
Outcomes	Diagnostic accuracy of biomarkers for detecting histological target conditions was the primary outcome measure and this was assessed by AUROC analysis and measures of sensitivity and specificity at biomarker predefined thresholds

Plants

Seed stocks	n/a
Novel plant genotypes	n/a
Authentication	n/a
