# Supplementary material for: Prospective validation of imaging and serum diagnostic biomarkers of steatohepatitis and fibrosis in MASLD: the LITMUS Imaging Study
Source: Nat Med. 2026 Jul 24;32(8):2959–69. doi: 10.1038/s41591-026-04496-2 (PMC13472969; doi:10.1038/s41591-026-04496-2)
Supplement: Supplementary file 3 [file 41591_2026_4496_MOESM3_ESM.pdf]

nature portfolio

Peer Review File

Prospective validation of imaging and serum diagnostic biomarkers of steatohepatitis and fibrosis in MASLD: The LITMUS Imaging Study

Corresponding Author: Professor Michael Pavlides

This file contains all reviewer reports in order by version, followed by all author rebuttals in order by version.

Version 0:

Reviewer comments:

Reviewer #1

(Remarks to the Author)
Dear authors,

Please find below my comments.

Major comments :
- This study reported the diagnostic performance of multiple imaging and serum biomarkers in the context of MASLD.
- I believe this study is a missed opportunity to study the impact of these multiple imaging and serum biomarkers on screening at-risk patients in comparison with recently reported guidelines (EASL-AASLD). Furthermore, a longitudinal follow-up and repeatability assessment of these biomarkers would have increased novelty and significance of the study.
- Specify the patient selection modalities: consecutive inclusion? stratification in the inclusion to ensure adequate representation of the target conditions? Provide the demographics of patients per inclusion site.
- Specify if quality criteria have been defined for biopsy samples and if the biopsy procedure was standardized across sites.
- VCTE: the type of probes should be specified
- The MRI sequences parameters should be provided
- This study aims to evaluate imaging biomakers for the detection of at-risk patients but biomarkers of potential significant value have not been included (IVIM, damping ratio, loss modulus, dispersion).
- Explain the rationale for using liver stiffness as a predictor of MASH. Inflammation has been reported as a confounding factor for fibrosis. The opposite is not true.
- Have you considered a 2-step strategy in predicting the target conditions?
- Have you considered iron overload as a confounding factor of the results. This is available with MRI PDFF.
- All thresholds used in this study should be justified. The threshold for MRE is surprising.
- Statistical analysis: justify the use of 0.80 for the AUC as the minimum acceptable diagnostic performance. Justify why you did not use accuracy metrics, which is more suited to a pre-defined threshold.

Minor comments :
- Detail the flowchart.
- Double-check legends of the figures including the axes.
- Improve esthetic finish of the figures.

Reviewer #2

(Remarks to the Author)
Dear Editor, i reviewed the submitted manuscript “Biomarkers for MASLD: Prospective evaluation of biomarkers for the diagnosis of Steatohepatitis and Fibrosis in the LITMUS Imaging Study”

General overview :

Major strengths
• Prospective relatively large multicenter cohorts with MASLD patients
• Central evaluation of diagnostic test and reference standard

Major weakness
• Relative new information on existent literature regarding the diagnostic performances of each test
• The large delay (6 months) between non invasive tests and reference standard should be considered as a potential source on limitation especially for assessment of steatosis and inflammation.

A. Summary of the key results
With this prospective multicenter study the authors aimed to evaluate and compare the
diagnostic performances of several noninvasive diagnostic biomarkers in patients with MASLD to grade and stage disease severity.

The main results if the study is that imaging and serum biomarkers showed complementary performance. Serum biomarkers tended to outperform imaging for at-risk MASH and Imaging techniques outperformed serum biomarkers for fibrosis assessment.

B. Originality and significance: if not novel, please include reference
Overall, this is a high-quality study that offers a valuable evaluation and comparison of several non-invasive biomarkers. Evaluating these tests within a single cohort is useful. However, the diagnostic performances of the biomarkers assessed (both for MASH and fibrosis) are already established in the literature. As such, the study is mainly confirmatory, even though previous data are not always derived from a single, unified cohort and not all prior studies have used a prospective design.

Some examples of original works, meta analysis and reviews are provided below :

https://pubmed.ncbi.nlm.nih.gov/38463540/
https://pubmed.ncbi.nlm.nih.gov/38548067/
https://pubmed.ncbi.nlm.nih.gov/38013143/
https://pubmed.ncbi.nlm.nih.gov/40212791/
https://pubmed.ncbi.nlm.nih.gov/41160822/
https://pubmed.ncbi.nlm.nih.gov/37121437/
https://www.nature.com/articles/s44355-025-00030-2

C. Data & methodology: validity of approach, quality of data, quality of presentation
Overall, in prospective diagnostic accuracy studies of MASLD, non-invasive biomarkers should be obtained as close as possible with the reference standard. A prolonged interval introduces biological variability in steatosis, activity and fibrosis and therefore requires explicit justification. The large delay (6 months) allowed between non-invasive test and reference standard should be considered as a potential source on limitation especially for assessment of steatosis and inflammation.

Histological assessement Line 288 : Please indicate whether any predefined quality criteria for liver biopsy specimens were included in the study.

Imaging Line 321 : i would suggest to provide details (as supplementary materials) on imaging data evaluation, namely ROI placement for PDFF and MRE. Also I would suggest to provide details on operator experience.

I would recommend including, as supplementary material, the sensitivity, specificity, and predictive values of the tests.

D. Appropriate use of statistics and treatment of uncertainties

The authors perform a weighted ROC analysis adjusted for age, sex, BMI, type 2 diabetes, and fibrosis, which is particularly important in light of the substantial heterogeneity across the available datasets for each biomarker. In this setting I would suggest providing more detailed information about the participating centres, including the distribution of enrolled patients across centres.

E. Conclusions: robustness, validity, reliability

Major strengths of the manuscript include the prospective design and the relatively large, multicentre cohort of MASLD patients, combined with centralized evaluation of both diagnostic tests and the reference standard.

Weaknesses that should be considered are:
• The study provides relatively limited new information beyond the existing literature regarding the diagnostic performance of each test with no changes in diagnostic pathway or patient management
• Data loss for head-to-head comparisons
• As already highlighted, the long delay (up to 6 months) between the non-invasive tests and the reference standard represents a potential limitation, particularly for the assessment of steatosis and inflammation.

F. Suggested improvements: experiments, data for possible revision
The long delay (up to 6 months) between the non-invasive tests and the reference standard represents a potential limitation, particularly for the assessment of steatosis and inflammation : in this context, whether patients underwent any specific interventions between the reference standard and the non-invasive test (e.g., lifestyle changes or initiation of pharmacological therapy) should be reported and considered in the data analysis, as those interventions may impact the results.

I would suggest providing more details about centres (for example distribution details of included patients per center), and to discuss the possible impact on provide results

Also I would suggest commenting on changes on diagnostic performances adjusted per country as reported in Supplementary Table S5 (I.e. reduction of diagnostic performance for MRE).

G. References: appropriate credit to previous work?
I acknowledge the importance of the present study and the challenges inherent in its design. In this setting please consider discussing the results of the Quid NASH cohort, a prospective multicenter study addressing a similar question in patients with type 2 diabetes:
https://pubmed.ncbi.nlm.nih.gov/38548067/

H. Clarity and context: lucidity of abstract/summary, appropriateness of abstract, introduction and conclusions

Some detailed comment :
Abstract

• Results pag 8 line 219-221 : "MAC for advanced fibrosis was significantly met by MRE (AUC 0.91) and AGILE3+ (AUC 0.84) and by MRE (AUC 0.91), VCTE-LSM (AUC 0.87), AGILE 3+ (AUC 0.89), and AGILE4 (AUC 0.88) for cirrhosis" : if these tests significantly exceed MAC please provide p value.

• Conclusion :

Line 223 : Serum biomarkers tended to outperform imaging for at-risk MASH : performances of imaging biomarkers for at-risk MASH should be provided in the abstract results.

Line224 : Imaging techniques outperformed serum biomarkers for fibrosis assessment : only combined markers (and not biological markers) performances for fibrosis are provided in the results .

Others :

Main text Introduction
Please consider add the sampling bias as potential additional limitation of biopsy in diagnosis/grading diffuse liver disease.

Discussion
The study provides relatively limited new information beyond the existing literature regarding the diagnostic performance of each test. What is the added value of these results in the diagnostic pathway or clinical management of MASLD patients?

Page 22 Line 530 to 533 : could you provide some hypothesis to this different results?

Page 22 line 547 : "disease activity can also fluctuate within a few weeks so the actual time window between liver biopsy and the acquisition of the non-invasive tests may impact the performance." : please refers to the comment regarding the maximum delay of 6 months between non invasive test and reference standard.

I would suggest provide and discuss details about centres (for example distribution of included patients).

Also I would suggest commenting on changes on diagnostic performances adjusted per country as reported in Supplementary Table S5 (I.e. reduction of diagnostic performance for MRE).

Tables

Table 1 : should be MAS score instead of NAS

Table 2 : I would suggest separating imaging, blood and composite scores.

Table 3 :
Could you provide a reference for the 8% PDFF threshold ?
typo in agile 3+ specificity table cell (missing parenthesis)

Figures

The lower limits of the confidence intervals in the blue histograms (imaging modalities) are hardly visible; enhancing their visibility would improve readability.

Reviewer #3

(Remarks to the Author)
This manuscript reports the prospective LITMUS Imaging Study, a large, international, multicentre diagnostic accuracy study evaluating a comprehensive panel of serum, imaging, and composite non-invasive biomarkers for the diagnosis of MASH and fibrosis in MASLD, using centrally read liver histology as the reference standard.
The scale of the study, the rigorous prospective design, centralised blinded assessment of biomarkers and histology, and the breadth of evaluated modalities represent a major strength and clearly distinguish this work from prior single-centre or retrospective studies. The results provide an important and, in some respects, sobering benchmark for the field.
Overall, this is a high-quality study that addresses a clinically and regulatory relevant question. However, several key issues regarding interpretation, framing, and clinical translation should be addressed to maximise its impact and suitability for Nature Medicine.

Major comments 1
One of the most important and informative findings of this study is that none of the evaluated non-invasive biomarkers significantly exceeded the prespecified minimally acceptable performance criterion (MAC; AUC >0.80) for either MASH or at-risk MASH, despite the scale, methodological rigor, and statistical power of the LITMUS Imaging Study.
This observation should not be framed as a shortcoming of individual biomarkers. Rather, it likely reflects fundamental biological and methodological limitations inherent to the current histology-based definitions of MASH and at-risk MASH. Specifically, key histological components used to define these entities—such as hepatocyte ballooning and lobular inflammation—are well recognised to exhibit substantial inter- and intra-observer variability, spatial sampling error, and short-term biological fluctuation. In this context, liver biopsy itself represents an imperfect reference standard, which may impose an upper bound on achievable diagnostic accuracy for any comparative non-invasive test.
The manuscript touches on these issues in the Discussion, but their implications remain under-emphasised. Given the clinical and regulatory relevance of MASH diagnostics, this finding warrants more explicit framing as a conceptual insight into the limits of current disease definitions, rather than as a negative diagnostic result.
The authors should strengthen this message in both the Introduction and the Conclusion, explicitly stating that the failure to surpass the MAC for MASH and at-risk MASH likely reflects intrinsic limitations of the histological reference standard and disease construct, and that this has important implications for biomarker development, validation, and regulatory qualification.

Major revision 2
While the use of AUC as the primary endpoint is appropriate for a diagnostic accuracy study, the manuscript relies almost exclusively on AUC to frame clinical relevance. However, AUC alone does not translate directly into real-world clinical decision-making, particularly in MASLD, where rule-in and rule-out strategies are central to patient management.
Although the authors report sensitivity and specificity at prespecified thresholds (Table 3), these results are not sufficiently integrated into the main narrative, leaving readers without clear guidance on how the biomarkers might be operationalised in practice. As presented, the clinical implications of the threshold-based analyses remain underdeveloped.
The manuscript would be strengthened by explicitly acknowledging the limitations of AUC as a sole metric and by embedding the threshold-based findings within pragmatic diagnostic workflows. For example, a conceptual discussion of sequential strategies—such as blood-based biomarkers for initial triage followed by elastography to confirm advanced fibrosis—would substantially improve clinical interpretability, even in the absence of additional formal analyses.

Major revision 3
The study population is restricted to patients undergoing clinically indicated liver biopsy in secondary and tertiary care centres. This design is appropriate for evaluating diagnostic accuracy against histology and enhances internal validity; however, it also enriches the cohort for more advanced disease and limits generalisability to lower-prevalence settings.
Although the authors note that the study was not designed for population screening, this distinction is not consistently foregrounded throughout the manuscript. Without clearer emphasis, there is a risk that readers may over-extrapolate the findings to primary care or screening contexts.
The manuscript would benefit from more explicit framing of the intended diagnostic context of use. Clearly stating—particularly in the Results section and/or figure legends—that these findings apply to biopsy-referred populations, and cautioning against extrapolation to screening or population-level case finding, would improve clarity and prevent misinterpretation.

Major revision 4
Several composite scores, including FAST, MAST, and cTAG, demonstrated lower diagnostic performance than reported in earlier studies. While the authors appropriately suggest that the prospective, multicentre design of LITMUS may reduce bias, the Discussion would benefit from a more explicit methodological comparison with prior literature.
In particular, clearer distinction between biomarker derivation studies and independent validation studies would help contextualise these discrepancies. Many earlier reports relied on threshold optimisation within development cohorts, smaller sample sizes, or narrower disease spectra, all of which can inflate apparent performance.

Framing the present results explicitly as robust external validation findings—and contrasting them with optimism-biased development studies—would strengthen the credibility of the conclusions and reinforce the value of LITMUS as a benchmark study rather than an outlier.

Major revision 5
The manuscript would benefit from a clearer articulation of the downstream implications of these findings for clinical practice and regulatory science.
The Results convincingly demonstrate that elastography-based biomarkers are suitable for ruling in advanced fibrosis and cirrhosis, whereas the role of non-invasive biomarkers for MASH activity assessment remains less well defined. Explicitly distinguishing biomarkers that appear ready for clinical implementation from those that should remain investigational would enhance the translational impact of the work.
In addition, while regulatory qualification is briefly mentioned, this aspect could be expanded. Clarifying how these data may inform biomarker qualification pathways with regulatory agencies, and what evidence gaps remain—such as outcome prediction or longitudinal responsiveness—would place the study in a broader translational and regulatory context.
Incorporating a schematic figure or boxed summary outlining a pragmatic diagnostic pathway informed by the study's findings could substantially improve accessibility and clinical relevance without requiring additional analyses.

Minor comments 1
The cohort is predominantly of White ethnicity (~94%). Please add a brief statement discussing the implications for generalisability to more ethnically diverse populations.

Minor comments 2
The permitted biopsy-to-imaging interval of up to six months may particularly affect activity-related endpoints. This should be highlighted more clearly as a limitation for MASH diagnostics.

Minor comments 3
Terminology (MAS, NAS, MASH-CRN) could be clarified early in the manuscript for consistency and readability.

Version 1:

Reviewer comments:

Reviewer #1

(Remarks to the Author)
I would like to thank the authors for resubmitting a revised manuscript. However, I do not believe the major comments of the reviewers have been adequately addressed. Although the study demonstrates rigorous methodology and major strengths, I align with Reviewer 2 regarding the limited novelty for publication in Nature Medicine.

Reviewer #2

(Remarks to the Author)
Dear authors,
Thank you for the revised version of the manuyscript.

Overall, the point-by-point replies are satisfactory, and the manuscript has improved in clarity and presentation.
I would suggest to define biopsy adequacy criteria explicitly including minimum biopsy length, portal tracts, fragmentation exclusions ,rejection criteria.

Reviewer #3

(Remarks to the Author)
Checked the revised manuscript. it is well corrected.

Open Access This Peer Review File is licensed under a Creative Commons Attribution 4.0 International License, which permits use, sharing, adaptation, distribution and reproduction in any medium or format, as long as you give appropriate credit to the original author(s) and the source, provide a link to the Creative Commons license, and indicate if changes were made.
In cases where reviewers are anonymous, credit should be given to 'Anonymous Referee' and the source.
The images or other third party material in this Peer Review File are included in the article's Creative Commons license, unless indicated otherwise in a credit line to the material. If material is not included in the article's Creative Commons license and your intended use is not permitted by statutory regulation or exceeds the permitted use, you will need to obtain permission directly from the copyright holder.
To view a copy of this license, visit https://creativecommons.org/licenses/by/4.0/

REVIEWER #1 (Remarks to the Author):

<u>Comment</u>

- This study reported the diagnostic performance of multiple imaging and serum biomarkers in the context of MASLD.
- I believe this study is a missed opportunity to study the impact of these multiple imaging and serum biomarkers on screening at-risk patients in comparison with recently reported guidelines (EASL-AASLD). Furthermore, a longitudinal follow-up and repeatability assessment of these biomarkers would have increased novelty and significance of the study.

<u>Our response.</u>

We thank the reviewer for the careful evaluation of our manuscript and the constructive comments. We agree that there is much still to learn about biomarker performance in MASLD/MASH. An increasing number of biomarkers have been proposed for patient assessment beyond those routinely included in clinical guidelines, yet many lack high-quality, independent evidence to support performance claims. Our study specifically addresses this important evidence gap: presenting prospectively acquired, robust and impartial data defining the diagnostic performance of multiple imaging and circulating biomarkers – encompassing tests that are well established and also cutting-edge technologies that are less widely available.

The current study was specifically designed to assess diagnostic accuracy of advanced imaging biomarkers. Recruitment was conducted in the secondary/tertiary care setting: the setting where MR imaging is available, and also the setting in which participants are recruited into therapeutic trials. The accepted histological reference standard for biomarker performance necessitates a highly invasive liver biopsy, making a truly unselected population sample to assess screening performance unethical, even in a high-risk group. We would however highlight that, whilst the pre-test probability may differ between primary care and secondary/tertiary care populations, the sensitivity/specificity and AUROC data presented in this paper remain relevant. In response to the reviewer's comment, we have now added text to the introduction to further clarify the purpose of this study. Please see our response to a similar point from reviewer 3 for details of the revisions.

We agree that longitudinal follow-up and repeatability assessment to address the prognostic context of use would be of interest but the reviewer will appreciate that, based on the well described slow natural history of MASLD, it takes many years to accrue sufficient clinical events in a prospectively recruited cohort. Follow-up is ongoing and we look forward to addressing that in future publications.

Comment

- Specify the patient selection modalities: consecutive inclusion? stratification in the inclusion to ensure adequate representation of the target conditions?

Our response

Participants undergoing diagnostic assessment for presumed MASLD were recruited consecutively at each site. We have clarified this in the methods section. We did not stratify at inclusion as we were aiming to have a distribution of the target conditions that is typical to that seen in clinical practice rather than have even distribution across target conditions. The latter would raise issues with spectrum bias when assessing diagnostic accuracy. We are confident that our cohort is highly representative of populations of patients seen in clinical practice in the secondary/tertiary care setting.

Comment

Provide the demographics of patients per inclusion site.

Our response

We thank the reviewer for raising this important point. As some of the centres contributed only small numbers of participants, we have included a table showing the characteristics of participants according to the country they were recruited from. This is now included as Supplementary Table S3.

Comment

- Specify if quality criteria have been defined for biopsy samples and if the biopsy procedure was standardized across sites.

Our response

Liver biopsy was performed at each site according to the standard local practice[1]. A standard operating procedure was followed for tissue sample handling with the FFPE blocks being transferred to a centralised biobank for processing/staining and digitisation. Biopsies were centrally read by expert members of the LITMUS Histopathology group and reported by consensus between two histopathologists. The adequacy of each biopsy was assessed prior to scoring.

Comment

- VCTE: the type of probes should be specified

Our response

We previously conducted an individual participant data meta-analysis in more than 5000 participants with paired liver stiffness measurements and biopsy where we showed that the choice of probe does not impact on the diagnostic performance of VCTE (see supporting table 22 in Mozes et al 2021 at: https://doi.org/10.1136/gutjnl-2021-324243). For this study the choice of probe was according to the automatic probe selection tool available on the latest versions of the fibroscan devices and data from measurements with the M (n=163), XL (n=154) probes and those where the choice of probe was not recorded (n=40) were analysed together.

Comment

The MRI sequences parameters should be provided

Our response

Full details of the MRI procedures in our study are available in the published study protocol (https://doi.org/10.1016/j.cct.2023.107352) which is cited in our paper. We have signposted our previous protocol publication more explicitly in the revised manuscript and added a supplementary methods section to give more details of the MRI protocols.

Comment

This study aims to evaluate imaging biomarkers for the detection of at-risk patients but biomarkers of potential significant value have not been included (IVIM, damping ratio, loss modulus, dispersion).

Our response

We agree with the reviewer that many other MRI parameters have been proposed. However, our aim was to provide validation for techniques that have sufficient prior data to support their use, or that are being commercialised currently. While a lot of techniques like IVIM, damping ratio, loss modulus and dispersions have shown some promise, they have not been sufficiently studied beyond a small number of cases. The current study remains the most comprehensive prospectively recruited biomarker performance study addressing advanced imaging biomarkers in MASLD/MASH.

Comment

Explain the rationale for using liver stiffness as a predictor of MASH. Inflammation has been reported as a confounding factor for fibrosis. The opposite is not true.

Our response

It is recognised that there is substantial co-linearity between the grade of steatohepatitis and the stage of fibrosis and risk of disease progression[2]. In addition, target conditions like “at-risk” MASH are themselves a composite of both steatohepatitis and fibrosis, and a number of composite tests

have been proposed that include elastography techniques to detect fibrosing steatohepatitis (eg FAST, MAST, etc). We agree with the reviewer that liver stiffness *per se* is not a direct measure of steatohepatitis activity but there is precedent for other indirect measures being widely adopted into clinical practice (eg FIB4 does not directly measure features of fibrosis). In addition, we wanted to ensure that no *a priori* assumptions introduced bias into the evaluation and so all biomarkers were assessed for all target conditions. This analysis has indeed yielded some very interesting results because despite the prior knowledge that liver stiffness may not be a very good biomarker for MASH, our study in fact found that MRE liver stiffness had the highest performance out of the imaging markers that were tested.

<u>Comment</u>

- Have you considered a 2-step strategy in predicting the target conditions?

<u>Our response</u>

We thank the reviewer for this suggestion and agree that examining how biomarkers would perform in 2-step strategies would be interesting. That analysis is outside the scope of this paper, where space is limited. It is something that we will look at in future studies. We have now added some examples of how our results can be used to constrict such 2-step strategies in response to this and a comment from reviewer 3.

<u>Comment</u>

- Have you considered iron overload as a confounding factor of the results. This is available with MRI PDFF.

<u>Our response</u>

We agree with the reviewer that MRI biomarkers may be subject to confounding by liver iron. However, analysis for confounding in our biomarkers was beyond the scope of this analysis.

<u>Comment</u>

- All thresholds used in this study should be justified. The threshold for MRE is surprising.

<u>Our response</u>

We examined thresholds that were previously reported in the literature. The details of the source publications are available in Supplementary Table 2.

Comment

- Statistical analysis: justify the use of 0.80 for the AUC as the minimum acceptable diagnostic performance. Justify why you did not use accuracy metrics, which is more suited to a pre-defined threshold.

Our response

When deciding about the metric to express the diagnostic accuracy of the biomarkers, we selected a priori the area under the ROC curve (AUC). The AUC summarizes test performance across all possible thresholds, rather than at arbitrary cut-offs, so it reflects the global discriminative ability. A single, interpretable metric for overall accuracy facilitates communication and decision-making.

This has been added to the Methods section in the manuscript

> “The AUC summarizes test performance across all possible thresholds, rather than at arbitrary cut-offs, enabling fairer evaluation and comparison of multiple biomarkers.”

In addition, we evaluated the performance of imaging markers using predefined thresholds, reported in the literature, for ruling in at-risk MASH or advanced fibrosis. The sensitivity and specificity estimates of various biomarkers at prespecified thresholds can be found in supplementary tables S7 and S8.

The minimum acceptable diagnostic performance was defined before data collection started in the LITMUS cohort, in a consensus-based process drawing on the expertise of clinicians, drug developers, biomarker developers and expert methodologists. The consensus was that a biomarker, to replace liver biopsy, should have a sufficiently high sensitivity and specificity relative to histology.

Comment

Minor comments :

- Detail the flowchart.
- Double-check legends of the figures including the axes.
- Improve esthetic finish of the figures.

Our response

We have checked our figures and their legends and have revised them to match the style of the journal.

REVIEWER#2 (Remarks to the Author):

Comment

• Prospective relatively large multicenter cohorts with MASLD patients

• Central evaluation of diagnostic test and reference standard

Our response

We agree with the reviewer that in terms of number of prospectively recruited participants, scope of imaging biomarkers being evaluated and the robustness of the evaluation our study is unique, and we are grateful to them for acknowledging these important features of this study.

Comment

Major weakness

• Relative new information on existent literature regarding the diagnostic performances of each test

Our response

The current study examines a comprehensive range of biomarkers – some longstanding and widely used in clinical practice, others much more novel. As the reviewer points out, there have been previous publications addressing performance of many of these tests. However, many of these studies have been of relatively small size, often recruited participants only in a single center, and have lacked the independence and scientific rigour of the current study. We respectively disagree with the reviewer's comment that no new information is presented in our paper. On the contrary, we believe that our study is the first to comprehensively examine the performance of both advanced imaging and circulating biomarkers in a truly prospective manner at multiple sites, and with centrally read histology as the reference standard and a defined chain of data custody to ensure a truly objective assessment. The fact that the data included here have been collected in rigorous standardised protocols is a major novelty of our study and this is elegantly summarised by reviewer 3 when they say:

"The scale of the study, the rigorous prospective design, centralised blinded assessment of biomarkers and histology, and the breadth of evaluated modalities represent a major strength and clearly distinguish this work from prior single-centre or retrospective studies. ***<u>The results provide an important and, in some respects, sobering benchmark for the field."</u>***

Comment

The large delay (6 months) between non invasive tests and reference standard should be considered as a potential source on limitation especially for assessment of steatosis and inflammation.

Our response

We thank the reviewer for raising this point and agree that biomarkers being compared to liver biopsy should be collected as close as possible to the time of biopsy. We would respectfully highlight that in fact 82% of participants had their imaging assessments and liver biopsy performed within 12-weeks of each other (mean 7 weeks). Whilst the protocol allowed a 6-month interval between biopsy and biomarker acquisition this was the pre-defined maximum allowable interval (chosen because it is also standard practice in regulatory approved clinical trials), the vast majority of participants had their assessments a lot closer to the time of biopsy.

To better communicate this, we have revised the relevant text in the second paragraph of our Results from: "*The average time interval between biopsy and blood sampling was 3.6 weeks (SD 4.8) and 7.0 weeks (SD 5.1) between biopsy and the first imaging assessment."*

To: "Even though a maximum 6-month interval was allowable between liver biopsy and biomarker evaluation, the majority of participants had assessments within a shorter interval (respectively, 82% and 75% of participants had imaging and blood-based biomarker acquisition within 12 weeks of liver biopsy). The mean interval between biopsy and blood sampling and imaging assessment was 3.6 weeks (SD 4.8) and 7.0 weeks (SD 5.1) respectively."

Therefore, we are confident that this potential source of bias was minimised as much as possible in this study and was in keeping with the rigorous standards applied to clinical trials.

Comment

A. Summary of the key results

With this prospective multicenter study the authors aimed to evaluate and compare the diagnostic performances of several noninvasive diagnostic biomarkers in patients with MASLD to grade and stage disease severity.

The main results if the study is that imaging and serum biomarkers showed complementary performance. Serum biomarkers tended to outperform imaging for at-risk MASH and Imaging techniques outperformed serum biomarkers for fibrosis assessment.

Our response

We agree that this is a good high-level overview of our study.

Comment

B. Originality and significance: if not novel, please include reference

Overall, this is a high-quality study that offers a valuable evaluation and comparison of several non-invasive biomarkers. Evaluating these tests within a single cohort is useful. However, the diagnostic performances of the biomarkers assessed (both for MASH and fibrosis) are already established in the literature. As such, the study is mainly confirmatory, even though previous data are not always derived from a single, unified cohort and not all prior studies have used a prospective design.

Some examples of original works, meta analysis and reviews are provided below :

https://pubmed.ncbi.nlm.nih.gov/38463540/
https://pubmed.ncbi.nlm.nih.gov/38548067/
https://pubmed.ncbi.nlm.nih.gov/38013143/
https://pubmed.ncbi.nlm.nih.gov/40212791/
https://pubmed.ncbi.nlm.nih.gov/41160822/
https://pubmed.ncbi.nlm.nih.gov/37121437/
https://www.nature.com/articles/s44355-025-00030-2

Our response

We thank the reviewer for his comment and for providing citations for some of our previous unrelated studies as well as the work of other groups. We agree that these publications evidence the substantial interest there is in developing non-invasive biomarkers. As addressed in detail above, the current study has substantial novelty, not least that it is founded on a new prospectively recruited cohort with advanced MR imaging – one of a very small number of MR cohorts in the world. We have addressed this in detail above, in our response to a previous comment from this Reviewer.

Comment

C. Data & methodology: validity of approach, quality of data, quality of presentation

Overall, in prospective diagnostic accuracy studies of MASLD, non-invasive biomarkers should be obtained as close as possible with the reference standard. A prolonged interval introduces biological variability in steatosis, activity and fibrosis and therefore requires explicit justification. The large delay (6 months) allowed between non-invasive test and reference standard should be considered as a potential source on limitation especially for assessment of steatosis and inflammation.

Our response

As explained above, this is a misunderstanding – 87% of participants had MR imaging and biopsy within 12-weeks (mean 7 weeks). Six months was the maximum permissible interval and was used as it matches the standard interval in drug trial protocols.

Comment

Histological assessment Line 288 : Please indicate whether any predefined quality criteria for liver biopsy specimens were included in the study.

Our response

Thank you for seeking clarification on this important point. Yes – as described above, liver biopsy was performed at each site according to the standard local practice[1]. A standard operating procedure was followed for tissue sample handling with the FFPE blocks being transferred to a centralised biobank for processing/staining and digitisation. Biopsies were centrally read by expert members of the LITMUS Histopathology group and reported by consensus between two histopathologists. The adequacy of each biopsy was assessed by the histopathologist prior to scoring.

Comment

Imaging Line 321 : i would suggest to provide details (as supplementary materials) on imaging data evaluation, namely ROI placement for PDFF and MRE. Also I would suggest to provide details on operator experience.

Our response

We thank the reviewer for raising this important point. As noted above in response to a similar question from Reviewer 1, technical details relating to the study MRI acquisition and analysis have previously been published in our protocol paper which is citated in the current manuscript[3].

To specifically address this, we have restructured the relevant section in our Methods to signpost our protocol paper more explicitly for more details on the technical MRI aspects of our study. We have also added a Supplementary Methods section in our Supplement where we provide a summary for the MRI data acquisition and analysis.

Comment

I would recommend including, as supplementary material, the sensitivity, specificity, and predictive values of the tests.

Our response

Thank you for seeking clarification on this. These details relating to the sensitivity and specificity of biomarkers at predefined thresholds are included in Table 3 of the main manuscript. In addition, comparisons of how such thresholds performed in our study compared to other studies in the literature are also already included as Supplementary tables S7 and S8.

Comment

D. Appropriate use of statistics and treatment of uncertainties

The authors perform a weighted ROC analysis adjusted for age, sex, BMI, type 2 diabetes, and fibrosis, which is particularly important in light of the substantial heterogeneity across the available datasets for each biomarker. In this setting I would suggest providing more detailed information about the participating centres, including the distribution of enrolled patients across centres.

Our response

This point was also raised by reviewer 1 and we have addressed it above by the addition of Supplementary Table S3 with details of participants per country of recruitment.

Comment

E. Conclusions: robustness, validity, reliability

Major strengths of the manuscript include the prospective design and the relatively large, multicentre cohort of MASLD patients, combined with centralized evaluation of both diagnostic tests and the reference standard.

Our response

Thank you for these positive comments. We agree with the summary of our studies strength. We further feel, that the weight of these strengths is such that this study should be considered as the benchmark for the evaluation of the diagnostic performance of MASLD biomarkers in comparison to liver biopsy.

Comment

Weaknesses that should be considered are:

• The study provides relatively limited new information beyond the existing literature regarding the diagnostic performance of each test with no changes in diagnostic pathway or patient management

• Data loss for head-to-head comparisons

• As already highlighted, the long delay (up to 6 months) between the non-invasive tests and the reference standard represents a potential limitation, particularly for the assessment of steatosis and inflammation.

Our response

These comments have already been addressed in detail as point-by-point responses above. We appreciate that, even though this is one of the largest and most highly characterised cohorts of its

kind, sample size becomes limited once the cohort is restricted to permit direct head to head comparisons. We have revised text in our discussion to reflect this – the relevant text now reads: *"Limitations also need to be acknowledged, in particular the limited sample size due to the study costs, especially when head to head comparisons were considered. "*

Comment

F. Suggested improvements: experiments, data for possible revision
The long delay (up to 6 months) between the non-invasive tests and the reference standard represents a potential limitation, particularly for the assessment of steatosis and inflammation: in this context, whether patients underwent any specific interventions between the reference standard and the non-invasive test (e.g., lifestyle changes or initiation of pharmacological therapy) should be reported and considered in the data analysis, as those interventions may impact the results.

Our response

We thank the reviewer for pointing out the lack of clarity regarding of the maximum allowable time interval and are pleased to confirm that the vast majority of cases had scan and biopsy within a tight time window with a mean interval of just 7 weeks. We feel we have addressed this in our responses above.

Comment

I would suggest providing more details about centres (for example distribution details of included patients per center), and to discuss the possible impact on provide results

Our response

This is similar to the point we have already addressed above. We include a supplementary table showing the characteristics of recruited participants per-country.

Comment

Also, I would suggest commenting on changes on diagnostic performances adjusted per country as reported in Supplementary Table S5 (I.e. reduction of diagnostic performance for MRE).

Our response

We thank the reviewer for this recommendation. We have added the following text to our discussion:

"The performance of biomarkers for the diagnosis of "at-risk MASH" in the country adjusted analysis (supplementary table S5) presents a very similar picture to the unadjusted analysis. MRE liver stiffness remains numerically the highest performing imaging biomarker albeit with a lower AUC of 0.69 (0.61-

0.75). Performance of NIS2+ remains stable and this biomarker remains best performing albeit below the MAC. "

Comment

G. References: appropriate credit to previous work?

I acknowledge the importance of the present study and the challenges inherent in its design. In this setting please consider discussing the results of the Quid NASH cohort, a prospective multicenter study addressing a similar question in patients with type 2 diabetes:

https://pubmed.ncbi.nlm.nih.gov/38548067/

Our response

We thank the reviewer for highlighting this valuable study. We have added some commentary relating to this study in our paper.

Comment

H. Clarity and context: lucidity of abstract/summary, appropriateness of abstract, introduction and conclusions

Some detailed comment :

Abstract

• Results pag 8 line 219-221: "MAC for advanced fibrosis was significantly met by MRE (AUC 0.91) and AGILE3+ (AUC 0.84) and by MRE (AUC 0.91), VCTE-LSM (AUC 0.87), AGILE 3+ (AUC 0.89), and AGILE4 (AUC 0.88) for cirrhosis" : if these tests significantly exceed MAC please provide p value.

• Conclusion :

Line 223 : Serum biomarkers tended to outperform imaging for at-risk MASH : performances of imaging biomarkers for at-risk MASH should be provided in the abstract results.

Line224 : Imaging techniques outperformed serum biomarkers for fibrosis assessment : only combined markers (and not biological markers) performances for fibrosis are provided in the results

Our response

We thank the reviewer for the very careful feedback of our abstract. We have taken this into account and also revised our abstract to satisfy the journal's word count requirements and style. Our abstract now reads as follows:

"There is a need for robust evaluations of non-invasive biomarkers for metabolic dysfunction–associated-steatotic-liver-disease (MASLD). This prospective multicentre study assessed diagnostic accuracy of imaging (including MR elastography liver stiffness [MRE]) and FibroScan™ [VCTE-LSM]), serum biomarkers (including NIS2+®) and composite scores (including AGILE3+ and AGILE4) for

centrally read steatohepatitis and fibrosis. An AUC>0.80 was set a-priori as the minimum acceptable performance criterion (MAC). Among 357 participants, fibrosis stages F0–F4 were present in 12%, 16%, 25%, 32%, and 15%, respectively. NIS2+® had the highest diagnostic accuracy among serum biomarkers for MASH (AUC 0.83) and at-risk MASH (AUC 0.82), although neither significantly exceeded the MAC (p=0.15 and p=0.19). Among imaging biomarkers, MRE showed the highest performance for MASH (AUC 0.71) and at-risk MASH (AUC 0.75), but these remained below the MAC. For fibrosis staging, performance was stronger: MRE met the MAC for advanced fibrosis (AUC 0.91; p<0.01), as did AGILE3+ (AUC 0.84; p=0.03). For cirrhosis, several biomarkers exceeded the MAC, including MRE (AUC 0.91; p<0.01), VCTE-LSM (AUC 0.87; p<0.01), AGILE3+ (AUC 0.89; p<0.01), and AGILE4 (AUC 0.88; p<0.01). Serum biomarkers tended to outperform imaging for identifying at-risk MASH, whereas elastography and composite scores demonstrated excellent accuracy for staging advanced fibrosis and cirrhosis."

Comment

Main text Introduction

Please consider add the sampling bias as potential additional limitation of biopsy in diagnosis/grading diffuse liver disease.

Our response

Our introduction focuses on the invasive nature and resource intensity disadvantages of liver biopsy. For economy of space the disadvantages of sampling bias as well as observer dependent bias are addressed in the discussion in text which currently reads:

"As described above, histology is an imperfect reference standard being subject to sampling variation as well as both inter- and intra-observer variation in reading[9]. With a theorized 90% sensitivity and 90% specificity for biopsy itself, it has been reported that the highest performance a comparative marker could reasonably achieve in a 40% prevalence setting is an AUC of 0.90.[55]"

Therefore, we believe that the issue of sampling variability in biopsy is already adequately addressed and adding a comment on sampling bias of biopsy to our introduction will be an unnecessary duplication.

Comment

Discussion

The study provides relatively limited new information beyond the existing literature regarding the diagnostic performance of each test. What is the added value of these results in the diagnostic pathway or clinical management of MASLD patients?

Our response

We have addressed the comment relating to the novelty of our study in our responses above. We have also revised the text in our manuscript to point out possible ways in which our results could help in the design of diagnostic pathways and clinical management of people with MASLD in our response to reviewer 3 below.

Comment

Page 22 Line 530 to 533 : could you provide some hypothesis to this different results?

Our response

This is likely related to potential biases from the study design of previous studies that were either smaller than our cohort or not prospective. We have added the following text to clarify.

"The study that described cTAG was a retrospective analysis of a prospectively recruited pooled cohort, while the study that described the deMILI NASH MRI score was a small single centre study. These factors, and the lack of central histological evaluation in the published studies are likely to have introduced bias that led to overestimation of the diagnostic performance."

Comment

Page 22 line 547 : "disease activity can also fluctuate within a few weeks so the actual time window between liver biopsy and the acquisition of the non-invasive tests may impact the performance." : please refers to the comment regarding the maximum delay of 6 months between non invasive test and reference standard.

Our response

As the reviewer rightly points out, we already acknowledge the issue relating to the potential bias that may arise due to the interval between biopsy and biomarker. We have already addressed in previous comments why we feel that this potential source of bias has been minimised as much as possible in our study.

Comment

I would suggest provide and discuss details about centres (for example distribution of included patients).

Our response

This point has been addressed in responses to the reviewer above.

<u>Comment</u>

Also I would suggest commenting on changes on diagnostic performances adjusted per country as reported in Supplementary Table S5 (I.e. reduction of diagnostic performance for MRE).

<u>Our response</u>

We have already addressed this in comments above and added new text to our discussion.

<u>Comment</u>

Table 1: should be MAS score instead of NAS

<u>Our response</u>

We thank the reviewer for picking this typographical error. This has been corrected in the revised manuscript

<u>Comment</u>

Table 2: I would suggest separating imaging, blood and composite scores.

<u>Our response</u>

We thank the reviewer for this suggestion. However, we feel that this is already done in our manuscript. The reporting of the biomarkers follows the same format throughout. Imaging biomarkers are reported first, followed by single blood markers, followed by multimarker scores followed by composite (blood and imaging) scores. The designation of each biomarker is included in Supplementary table S1 and is not repeated in each table to avoid duplication.

<u>Comment</u>

Table 3:

Could you provide a reference for the 8% PDFF threshold?

<u>Our response</u>

The citations for all the biomarker thresholds that we used are already included in Supplementary Table S2.

<u>Comment</u>

typo in agile 3+ specificity table cell (missing parenthesis)

Our response

We thank the reviewer for picking this typographical error. This has been corrected in the revised manuscript

Comment

Figures

The lower limits of the confidence intervals in the blue histograms (imaging modalities) are hardly visible; enhancing their visibility would improve readability.

Our response

We thank the reviewer for pointing this out. The figure has been redrawn with a different colour scheme for the error bars to improve readability and visibility.

REVIEWER #3 (Remarks to the Author):

Comment

This manuscript reports the prospective LITMUS Imaging Study, a large, international, multicentre diagnostic accuracy study evaluating a comprehensive panel of serum, imaging, and composite non-invasive biomarkers for the diagnosis of MASH and fibrosis in MASLD, using centrally read liver histology as the reference standard.

The scale of the study, the rigorous prospective design, centralised blinded assessment of biomarkers and histology, and the breadth of evaluated modalities represent a major strength and clearly distinguish this work from prior single-centre or retrospective studies. The results provide an important and, in some respects, sobering benchmark for the field.

Overall, this is a high-quality study that addresses a clinically and regulatory relevant question. However, several key issues regarding interpretation, framing, and clinical translation should be addressed to maximise its impact and suitability for Nature Medicine.

Our response

We are grateful to the reviewer for their positive comments agree that this study should be regarded as the benchmark for the diagnostic accuracy of imaging biomarkers in MASLD. We are grateful for the constructive feedback.

Comment

Major comments 1

One of the most important and informative findings of this study is that none of the evaluated non-invasive biomarkers significantly exceeded the prespecified minimally acceptable performance criterion (MAC; AUC >0.80) for either MASH or at-risk MASH, despite the scale, methodological rigor, and statistical power of the LITMUS Imaging Study.

This observation should not be framed as a shortcoming of individual biomarkers. Rather, it likely reflects fundamental biological and methodological limitations inherent to the current histology-based definitions of MASH and at-risk MASH.

Specifically, key histological components used to define these entities—such as hepatocyte ballooning and lobular inflammation—are well recognised to exhibit substantial inter- and intra-observer variability, spatial sampling error, and short-term biological fluctuation. In this context, liver biopsy itself represents an imperfect reference standard, which may impose an upper bound on achievable diagnostic accuracy for any comparative non-invasive test.

The manuscript touches on these issues in the Discussion, but their implications remain under-emphasised. Given the clinical and regulatory relevance of MASH diagnostics, this finding warrants more explicit framing as a conceptual insight into the limits of current disease definitions, rather than as a negative diagnostic result.

The authors should strengthen this message in both the Introduction and the Conclusion, explicitly stating that the failure to surpass the MAC for MASH and at-risk MASH likely reflects intrinsic limitations of the histological reference standard and disease construct, and that this has important implications for biomarker development, validation, and regulatory qualification.

Our response

We agree with reviewer that the message relating the fundamental weaknesses of current MASH histological definitions were not well articulated in our paper. We have revised the relevant paragraph in our discussion to address this.

The relevant paragraph now reads:

"Our data suggests that measures of disease activity that comprise biologically plausible circulating markers of inflammation are promising for use in detecting MASH. It was therefore surprising that despite our methodological rigor none of the biomarkers met the MAC for MASH and at-risk MASH. We believe that this reflects inherent bias in the current histological definition of MASH. It is well established that the histological features of ballooning and lobular inflammation are subject to substantial observer-dependent variability[9]. Furthermore, disease activity is known to be heterogenous across the liver, leading to biopsy sampling error, and can also fluctuate with time. These factors may contribute to variation in the approximation of histology to the true status of the

liver as a whole but, as histology is the reference standard, the performance of the blood-based or imaging biomarker would still be penalised, even if it were a more faithful approximation in reality."

Comment

Major revision 2

While the use of AUC as the primary endpoint is appropriate for a diagnostic accuracy study, the manuscript relies almost exclusively on AUC to frame clinical relevance. However, AUC alone does not translate directly into real-world clinical decision-making, particularly in MASLD, where rule-in and rule-out strategies are central to patient management.

Although the authors report sensitivity and specificity at prespecified thresholds (Table 3), these results are not sufficiently integrated into the main narrative, leaving readers without clear guidance on how the biomarkers might be operationalised in practice. As presented, the clinical implications of the threshold-based analyses remain underdeveloped.

The manuscript would be strengthened by explicitly acknowledging the limitations of AUC as a sole metric and by embedding the threshold-based findings within pragmatic diagnostic workflows. For example, a conceptual discussion of sequential strategies—such as blood-based biomarkers for initial triage followed by elastography to confirm advanced fibrosis—would substantially improve clinical interpretability, even in the absence of additional formal analyses.

Our response

Thank you for this suggestion. We have added the following paragraph to our discussion to elaborate on the analysis relating to cut-offs.

"Our analysis relating to biomarker performance at predefined thresholds produced some results worth further commentary. For at risk MASH, some biomarkers have complementary performance and could potentially be combined in 2 step approaches. For example, tests with high sensitivity (like PDFF at a threshold of 8% (sensitivity 0.89), or CAP at a threshold of 280dB/m (sensitivity 0.92) can be applied first, followed by tests with high specificity such as MRE at 3.3kPa (sensitivity 0.76) or FAST at a threshold of 0.67 (specificity 0.86) or MAST at threshold 0.242 (specificity 0.91). Such approaches could be used in screening for clinical trial recruitment to help reduce screen failure rates. With increasing numbers of approved pharmacotherapies, such approaches can also help with selecting patients suitable for treatment.

The results for the performance of biomarkers at prespecified thresholds for advanced fibrosis, reflect the results of the AUC analysis and confirm that elastography techniques and composites scores that include elastography achieve high sensitivity and specificity. "

Comment

Major revision 3

The study population is restricted to patients undergoing clinically indicated liver biopsy in secondary and tertiary care centres. This design is appropriate for evaluating diagnostic accuracy against histology and enhances internal validity; however, it also enriches the cohort for more advanced disease and limits generalisability to lower-prevalence settings.

Although the authors note that the study was not designed for population screening, this distinction is not consistently foregrounded throughout the manuscript. Without clearer emphasis, there is a risk that readers may over-extrapolate the findings to primary care or screening contexts.

The manuscript would benefit from more explicit framing of the intended diagnostic context of use. Clearly stating—particularly in the Results section and/or figure legends—that these findings apply to biopsy-referred populations, and cautioning against extrapolation to screening or population-level case finding, would improve clarity and prevent misinterpretation.

Our response

Thank you for highlighting this point. We agree that this is the case and have revised the last paragraph of our introduction to reflect this. The new text now reads:

"A wide range of biomarkers based on serum or imaging tests, including elastography or combinations of the two, have been described. Vibration-controlled transient elastography (VCTE) is one of the most extensively studied, while magnetic resonance elastography (MRE) and other quantitative magnetic resonance (MR) techniques that do not require additional hardware have also been proposed. Whilst a number of studies have evaluated individual biomarkers or a limited number of biomarkers in small cohorts, evaluation and validation of multiple imaging and blood-based biomarkers in a large, prospective multicentre study is lacking. Our study specifically addresses this important evidence gap: presenting prospectively acquired, robust and impartial data defining the diagnostic performance of multiple imaging and circulating biomarkers – encompassing tests that are well established and also cutting-edge technologies that are less widely available. The main aim of the LITMUS Imaging Study was to robustly assess and independently evaluate the performance of MR, ultrasound-based imaging and elastography biomarkers for the accurate grading and staging of MASLD. A secondary aim was to compare the performance of imaging biomarkers to serum biomarkers. This study was specifically designed to assess the diagnostic accuracy of non-invasive biomarkers in people with MASLD that have already been referred to secondary / tertiary care; and the results should not be extrapolated to primary care population screening, where disease prevalence will be lower."

We have also revised the first paragraph of our discussion to stress this point.

The revised text now reads:

"In this diagnostic accuracy study of multiple imaging and serum biomarkers, performance was assessed for the diagnosis of four histologically-defined target conditions addressing disease activity (MASH; at-risk MASH: MAS ≥4 with F≥2) and staging of hepatic fibrosis (advanced fibrosis, and cirrhosis). The population under evaluation were people with MASLD that were already assessed in secondary and tertiary care centres and were referred for biopsy for disease evaluation; our findings should not be extrapolated to other settings like screening in primary care and in type 2 diabetes. We would however highlight that, whilst the pre-test probability may differ between primary care and secondary/tertiary care populations, the sensitivity/specificity and AUROC data presented in this paper remain relevant."

Comment

Major revision 4

Several composite scores, including FAST, MAST, and cTAG, demonstrated lower diagnostic performance than reported in earlier studies. While the authors appropriately suggest that the prospective, multicentre design of LITMUS may reduce bias, the Discussion would benefit from a more explicit methodological comparison with prior literature.

In particular, clearer distinction between biomarker derivation studies and independent validation studies would help contextualise these discrepancies. Many earlier reports relied on threshold optimisation within development cohorts, smaller sample sizes, or narrower disease spectra, all of which can inflate apparent performance.

Framing the present results explicitly as robust external validation findings—and contrasting them with optimism-biased development studies—would strengthen the credibility of the conclusions and reinforce the value of LITMUS as a benchmark study rather than an outlier.

Our response

We thank the reviewer for this suggestion. A similar point was also raised by Reviewer 2 with regards to cTAG and deMILI – We have already included a more detailed comparison between the published literature and our results in relation to these two biomarkers in response to that comment.

To address this comment in relation to FAST and MAST and to place our study more firmly as the benchmark rather than an outlier, we have added the following text in the discussion:

"The performance of composite scores in our study was in general lower than previously described. A previous metanalysis reported a summary AUROC of 0.79 (0.77-0.81) for FAST[43] (cf AUC of 0.73 in this study). The index studies in that meta-analysis were in many cases retrospective and included a

variety of populations (e.g. screening population in the AURORA clinical trial[44], bariatric surgery cohorts[45]). These factors are likely to have introduced bias that inflated the previously reported performance of FAST. MAST was previously evaluated in small studies from one or two centres that reported AUCs of 0.80 (0.72-0.89)[46] and 0.93 (0.88-0.97)[37] respectively (cf AUC of 0.78 in this study). In a cohort with type 2 diabetes, FAST (AUC 0.81) and MAST (AUC 0.79) were also reported to have higher performance for at risk MASH[47] but results are difficult to compare with our study due to differences in the population being evaluated. In comparison to previous studies that are subject to possible bias from study design, heterogeneity of the included population and lack of multicentre prospective validation, we believe that our study provides robust external validation for biomarker performance that is closer to the true biomarker performance."

To make it more explicit that we regard this study as the benchmark for the evaluation of MASLD biomarkers we have restructured our discussion and added this as the second paragraph:
"*The scale of the study in its geographical distribution, number of recruiting centres, the size of the recruited cohort, central histology evaluation and the scope of biomarkers assessed (both the range of MR and ultrasound modalities alongside numerous blood-based biomarkers) is unique. These key strengths led to minimisation of potential bias, in contrast to previous studies, and therefore our results represent a truly robust external validation for biomarker performance."*

Comment

Major revision 5
The manuscript would benefit from a clearer articulation of the downstream implications of these findings for clinical practice and regulatory science.
The Results convincingly demonstrate that elastography-based biomarkers are suitable for ruling in advanced fibrosis and cirrhosis, whereas the role of non-invasive biomarkers for MASH activity assessment remains less well defined. Explicitly distinguishing biomarkers that appear ready for clinical implementation from those that should remain investigational would enhance the translational impact of the work.
In addition, while regulatory qualification is briefly mentioned, this aspect could be expanded. Clarifying how these data may inform biomarker qualification pathways with regulatory agencies, and what evidence gaps remain—such as outcome prediction or longitudinal responsiveness—would place the study in a broader translational and regulatory context.

Incorporating a schematic figure or boxed summary outlining a pragmatic diagnostic pathway informed by the study's findings could substantially improve accessibility and clinical relevance without requiring additional analyses.

Our response

We have revised our discussion as follows to address this point:

"*Our study has important implications for clinical practice indicating that MRE and Agile3+ can be used to positively rule-in advanced fibrosis and cirrhosis, while VCTE-LSM and Agile4 can be used to rule-in cirrhosis. Therefore, these biomarkers can be used as confirmatory tests for disease severity once patients are referred to secondary / tertiary care. Biomarkers that did not meet the MAC for fibrosis assessment should remain investigational and should be examined in different contexts (e.g. screening in primary care or in diabetes clinics). The fact that none of the biomarkers met the MAC for MASH indications raises serious concerns about the validity of MASH as a target condition. The use of MASH as part of endpoint definitions in clinical trials and as a potential feature to be used when selecting patients for treatment with approved pharmacotherapies should therefore be re-examined in view of our results.*

While our study provides useful data on biomarker performance, we are limited by the use of histological target conditions. Establishing the performance of biomarkers for the prognosis of adverse outcomes would be more clinically relevant. The longitudinal data being generated within the Europe SLD (MASLD) Registry will be an important asset for assessing the prognostic value of biomarkers and addressing this evidence gap. Furthermore, future studies are also needed to address the performance of biomarkers in other contexts like monitoring or assessing therapeutic response. Lastly, future health economics studies should evaluate whether the superior performance of the more expensive tests like MR biomarkers and proprietary serum-based tests translate into cost-effectiveness."

Comment

Minor comments 1

The cohort is predominantly of White ethnicity (~94%). Please add a brief statement discussing the implications for generalisability to more ethnically diverse populations.

Our response

We agree with the reviewer that this is an important point to make. We have added the following text to our discussion:

“Lastly, our cohort is predominantly made up of people of White ethnicity (94%). Our results may therefore not be representative of biomarker performance in other ethnic groups or diverse populations. “

Comment

Minor comments 2

The permitted biopsy-to-imaging interval of up to six months may particularly affect activity-related endpoints. This should be highlighted more clearly as a limitation for MASH diagnostics.

Our response

We have addressed this it in our responses above to reviewers 1 and 2.

Comment

Minor comments 3

Terminology (MAS, NAS, MASH-CRN) could be clarified early in the manuscript for consistency and readability.

Our response

We have checked the manuscript throughout and changed the terminology from NAS to MAS in table 1 – this was also picked up by reviewer 2. We also refer to NASH in the name of the deMILI biomarker (NASH-MRI), but as this refers to the biomarker rather than the condition, we have kept the terminology as in the originally submitted manuscript.

References

1. Hardy T, Wonders K, Younes R, et al. The European NAFLD Registry: A real-world longitudinal cohort study of nonalcoholic fatty liver disease. Contemp Clin Trials 2020;98:106175.
2. Brunt EM, Kleiner DE, Wilson LA, et al. Improvements in Histologic Features and Diagnosis Associated With Improvement in Fibrosis in Nonalcoholic Steatohepatitis: Results From the Nonalcoholic Steatohepatitis Clinical Research Network Treatment Trials. Hepatology 2019;70:522-531.
3. Pavlides M, Mózes FE, Akhtar S, et al. Liver investigation: Testing marker utility in steatohepatitis (LITMUS): Assessment & validation of imaging modality performance across the NAFLD spectrum in a prospectively recruited cohort study (the LITMUS imaging study): Study protocol. Contemporary Clinical Trials 2023;134:107352.

<u>Response to reviewer comments</u>

Reviewer #1:
Remarks to the Author:
I would like to thank the authors for resubmitting a revised manuscript. However, I do not believe the major comments of the reviewers have been adequately addressed. Although the study demonstrates rigorous methodology and major strengths, I align with Reviewer 2 regarding the limited novelty for publication in Nature Medicine.
Our response
We thank the reviewer for their feedback. We note that no more specific points are raised for further clarification. As we have set out in our previous responses we respectfully disagree with the view that our study lacks novelty. Furthermore, we also note that reviewer 2 no longer has concerns regarding the novelty of our study after the revision.

Reviewer #2:
Remarks to the Author:
Dear authors,
Thank you for the revised version of the manuscript.
Overall, the point-by-point replies are satisfactory, and the manuscript has improved in clarity and presentation.
I would suggest to define biopsy adequacy criteria explicitly including minimum biopsy length, portal tracts, fragmentation exclusions ,rejection criteria.

Our response
We are pleased that the reviewer finds that the revision has improved the clarity and presentation of our work.
The reporting of liver biopsies and assessment of histological sample adequacy in our study was according to the position statement of the International MASLD Pathology group[1] (https://pubmed.ncbi.nlm.nih.gov/41072805/)
We have added the following statement in our methods to clarify this point:
"A liver biopsy with no more than 3 fragments, at least 1.5 cm in length and containing at least 6 portal tracts was considered adequate for the diagnosis of MASLD/MASH by the LITMUS Histopathology Group (LHG). In very few cases which did not meet the quality recommendations above, if all histological features for diagnosing MASH, according to the accepted minimum histological criteria, were clearly seen in a biopsy specimen despite suboptimal quality, the specimen was considered adequate. The above is in keeping with the consensus for the standardized application of histological grading and staging systems in MASH by the International MASLD Pathology group[1], whose members include the majority of the LHG. Biopsies that did not meet the set adequacy criteria and did not display the minimal histological features for diagnosing MASH were rejected."

Reviewer #3:
Remarks to the Author:
Checked the revised manuscript. it is well corrected.
Our response
We thank the reviewer for taking the time to check our revised manuscript and we are delighted that they find it well corrected.

References

1. Lackner, C., *et al.* Consensus position statements for the standardized application of histological grading and staging systems in MASH clinical trials. *J Hepatol* **84**, 693-701 (2026).
